# Functional nanoparticle-enabled non-genetic neuromodulation

**DOI:** 10.1186/s12951-023-02084-x

**Published:** 2023-09-07

**Authors:** Zhimin Zhang, Yanling You, Min Ge, Han Lin, Jianlin Shi

**Affiliations:** 1 Shanghai Institute of Ceramics Chinese Academy of Sciences, Research Unit of Nanocatalytic Medicine in Specific Therapy for Serious Disease, Chinese Academy of Medical Sciences, Shanghai, 200050 People’s Republic of China; 2 Shanghai Tenth People’s Hospital, Shanghai Frontiers Science Center of Nanocatalytic Medicine, School of Medicine, Tongji University, Shanghai, 200331 People’s Republic of China; 3 Center of Materials Science and Optoelectronics Engineering, University of Chinese Academy of Sciences, Beijing, 100049 People’s Republic of China

**Keywords:** Field response, Nanoparticles, Neuromodulation, Ion channels, Neurological diseases

## Abstract

Stimulating ion channels targeting in neuromodulation by external signals with the help of functionalized nanoparticles, which integrates the pioneering achievements in the fields of neurosciences and nanomaterials, has involved into a novel interdisciplinary field. The emerging technique developed in this field enable simple, remote, non-invasive, and spatiotemporally precise nerve regulations and disease therapeutics, beyond traditional treatment methods. In this paper, we define this emerging field as nano-neuromodulation and summarize the most recent developments of non-genetic nano-neuromodulation (non-genetic NNM) over the past decade based on the innovative design concepts of neuromodulation nanoparticle systems. These nanosystems, which feature diverse compositions, structures and synthesis approaches, could absorb certain exogenous stimuli like light, sound, electric or magnetic signals, and subsequently mediate mutual transformations between above signals, or chemical reactions, to regulate stimuli-sensitive ion channels and ion migrations which play vital roles in the nervous system. We will also discuss the obstacles and challenges in the future development of non-genetic NNM, and propose its future developments, to add the further progress of this promising field.

**Graphical Abstract:**

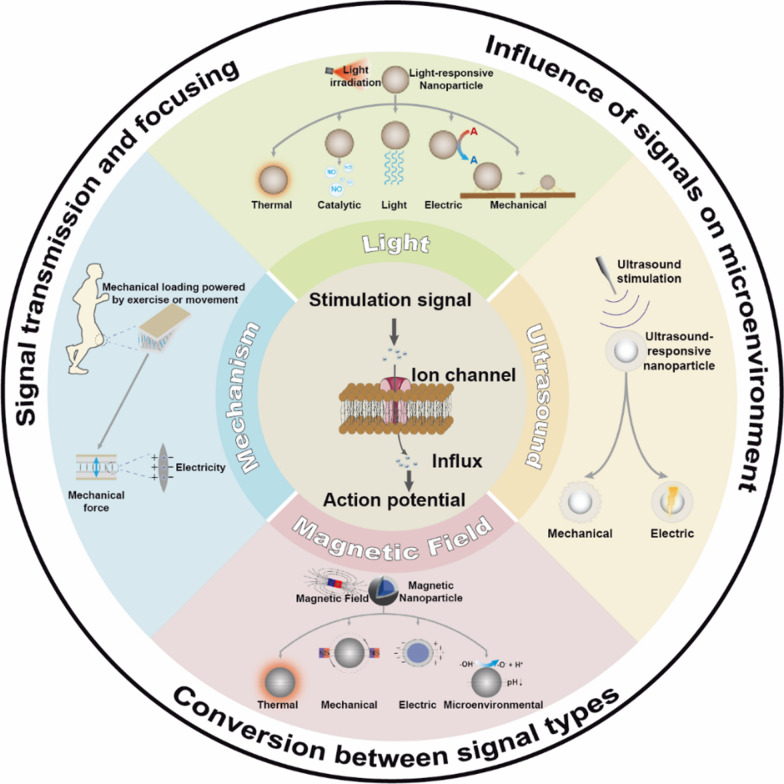

## Introduction

Nervous system plays an indispensable role in the normal and healthy running of human body. Neuronal diseases are the leading cause of disability and the second leading cause of death worldwide [1]. Globally, the burden of neurological diseases has been insufficient to meet the UN Sustainable Development Goal targets by 2030 [2]. Traditional treatments of neurological illnesses are rather unsatisfactory, which in most cases only perform limited effectiveness for certain symptoms without stopping the deterioration of the conditions while result in side-effects, especially in curing common neurodegenerative disorders like Parkinson’s disease (PD) [3, 4], Alzheimer’s disease (AD) [5], Huntington disease (HD) [6], Amyotrophic lateral sclerosis (ALS) [7] and Spinal muscular atrophy (SMA) [8]. The emerging strategies and techniques such as stem cell therapy would potentially help to cure neurological deceases, but there is a considerable distance to go for clinical success [9]. For example, the clinical stem cell therapy has been held back by challenges in avoiding tumour formation and influencing the environment of lesion areas [10]. Thus, a safe, practical and radical cure for neurological diseases is urgently needed.

As the most sophisticated component of the body, nervous system relies on action potentials to deliver messages and regulate physiological activities [11, 12]. These potentials are directly or indirectly triggered by various ion channels, which mainly refer to channel proteins on cytomembrane to play an important role in neuronal functions by controlling ion flows for signal delivery, demonstrating a hot spot in therapeutic researches of neurological disorders and the major modulating sites of neuromodulation [13, 14]. As such, ion channels have evolved various mechanisms through which the ionic conductance can be turned on and off, a process known as gating, in response to various cellular signals [15]. The stimuli for gating can be diverse such as thermally sensitive transient receptor potential (TRP) [16, 17] channels and pressure-sensing Piezo [18] channels, and many ion channels contain modular domains that directly sense the stimulus and then modulate the pore through domain−domain interactions [19–22]. These ion channel families are specifically sensitive to exogenous signals like light irradiation, sound wave, electric current, thermal effect, mechanical force and magnetic field, making it possible to achieve neuromodulation by applying external signals to cure related channelopathies. However, whole-body applications of external signals lack efficiency and targeting ability, traditional local stimulation method usually requires complicated and invasive procedures or implants, and certain signals as ultraviolet (UV) light are impractical to be directly utilized due to their potential harmful or poisonous side effects [23]. Evidently, the targeted regulation of neurons through non-invasive external field stimulation would be a therapeutic strategy with great potential for clinical translation [24].

Nanoparticles or nanostructures, performing large surface area, good biocompatibility and diverse biologic effects, will emerge as the most representative candidates for versatile and immense biomedical applications to benefit the health of human beings [25]. The elaborately engineered nanoparticles convert the external stimuli including light, sound, electricity, heat, force and magnetism into specific responses or products such as reactive hyperthermia [26, 27], reactive oxygen species (ROS) [28, 29], or electric effect [30, 31]. The most representative examples of these stimuli-responsive nanoparticles should be nanoscale drug carriers that perform sensitivity to internal stimulation elements like pH and redox potentials to achieve precise spatiotemporal drug release [32]. Especially, nanoparticles in neuromodulation are based on a particular sensitivity to exogenous physical signals rather than endogenous stimuli, which can trigger the activation or inhibition of certain types of ion channels in a remote and non-invasive manner. Original research of this category could date back to earlier than one decade ago, like the method reported by Huang in 2010 to activate temperature-sensitive ion channels by applying nanoparticles performing magnetic field-generated heat [33]. With the rapid growth of nanotechnology and neurobiology, increasing numbers of nanoparticles sensitive to external signals and ion channels modulating neuronal activities have been reported, making this sort of studies an emerging systematic field. These nanoparticles, including photothermal nanoparticles [23, 34–37], upconversion nanoparticles (UCNPs) [38–40], magnetic nanoparticles [33, 41–43], ultrasound (US) -mechanical nanoparticles [44], piezoelectric nanoparticles [24, 45], et al., though seemingly not accomplishable to be bracketed as a same category according to their composition or structure, all have pivotal roles in transforming exogenous stimuli into signals inside the body, which directly modulates the behaviours of neuromodulation-relevant ion channels by different external field stimulus responses, providing material science support for neuromodulation. We defined these non-invasive, biocompatible and precise neuromodulation strategies, enabling the stimulation of ion channels by using external signal-responsive nanoparticles, as nano-neuromodulation (NNM) [46–48].

Researches of NNM could be divided into two branches, genetic and non-genetic approaches, the development of which used to be uneven to a considerable extent. Before the breakthrough of the discovery and research of stimuli-sensitive ion channels, genetic approaches, especially optogenetics, which achieves stimuli-mediated neuromodulation down to the cellular level with the help of genetic engineering methods to combine stimuli-responsive proteins with target cells, have dramatically grown to be a precise alternative to traditional neurological treatments [49]. However, the require of modifying genes before targeting cells and unstable expression of the modified genes greatly challenges the practical use of genetic approaches in human body, making it necessary to pay more attention to non-genetic techniques, that is, non-genetic nano-neuromodulation (non-genetic NNM) [34, 39, 49–51]. And with the discovery of increasing number of ion channels (such as TRPV1, Piezo1 et al.) sensitive to external signals, it became possible to regulate neuro activities directly using exogenous physical stimuli, which goes through functional nanoparticle-mediated transmission and/or conversion to be precisely applied to these ion channels, without the accompany of extra stimuli-responsive proteins, hence eliminates the need of gene editing, overcoming the shortcomings of genetic approaches and becomes a new hotspot of NNM.

**Fig. 1:**
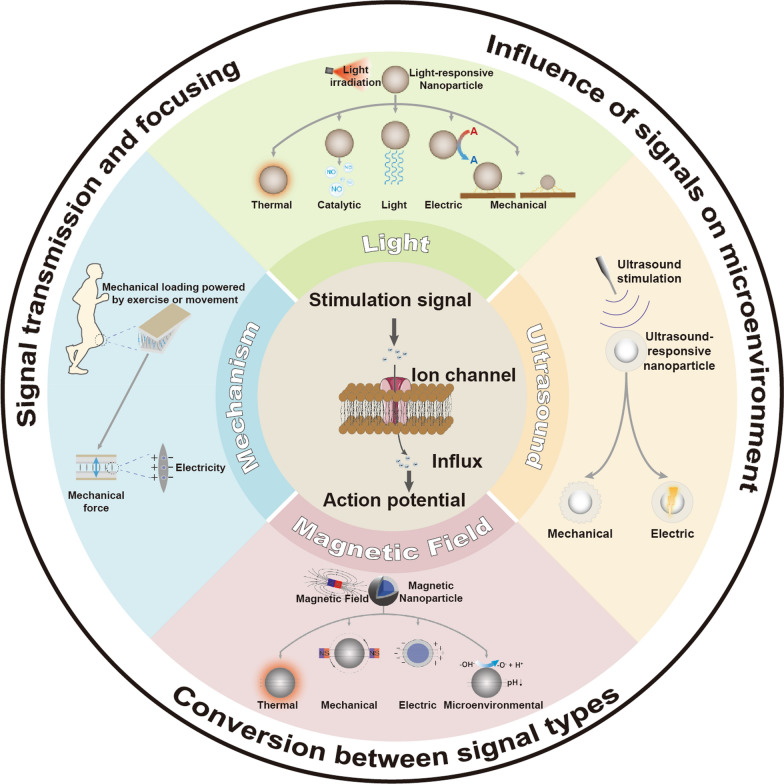
Schematic illustration of non-genetic nano-neuromodulation achieved by transferring or transforming exogenous acoustic, light, force, thermal, electric, and magnetic signals through functional nanoparticles and making them stimulate the ion channels on the surface of nerve cells, resulting in the influx of ions regulating membrane potential like Ca^2+^, and finally triggering the action potentials

In this review, we classified non-genetic NNM into the following sections based on the type of exogenous modulating signals, such as light, magnetic field, ultrasound and mechanical force response modes, and summarized the progress of them. We focused on the composition, structure and design of the nanoparticles enabling non-genetic neuromodulation, and analyzed the signal transmission/transformation process mediated, the ion channel stimulated, the types of neural activity regulated, the neurological diseases with potential efficacy, and the intrinsic mechanisms of the regulatory processes. In addition, we summarized the current state of research, unresolved issues and potential challenges for clinical applications in this emerging field and forecasted its future development directions as remotely controllable, spatiotemporally precise and multi-signal responsive non-genetic NNM (Fig. 1).

## Light-responsive neuromodulation

The application of light signals like near-infrared spectrum (NIR) in regulating neuronal activities initiated earlier than NNM, the major representation of which refers to optogenetics. As a cutting edge technique which aims at controlling photoactivatable opsins to stimulate activities of nerve cells and treat related diseases, optogenetics yet had been blocked from clinic application for a long time, the major obstacle of which should be the expressing instability and potential risk of exogenous genes and the weak penetration of optical spectra [52]. However, these disadvantages have been greatly eased by functional nanoparticles, especially the drawbacks of optical spectra, the mismatch between penetration and triggering ability of which could be eliminated with finesse by some practical conversion processes like upconversion from NIR to visible light [53] and photothermal effect [36] by virtue of certain nanoparticles. Hence, the combination of functional nanoparticles and optogenetics gave birth to nano-enabled genetic light-responsive neuromodulation. Not only the genetic branch, thanks to the high penetration depth of NIR and the convenient transformation between NIR and other signal types mediated by nanoparticles, light-responsive non-genetic NNM, which shows better performance and biocompatibility compared to commonly-used implant methods based on the electrical signal, have developed into a variety of modulation strategies involving abundant types of nanoparticles and ion channels, and has become the most developed section of non-genetic NNM.

### Photothermal-responsive neuromodulation

Base on the fact that temperature perception in living organisms is crucial to avoid danger and maintain internal homeostasis, the thermo-response approach should be an important entry point for non-genetic NNM, as more and more thermol-signal sensitive ion channels have been reported, such as the TRP [35] and TREK [34] families, which make it possible to establish thermol-responsive neuromodulation [54, 55]. However, due to the lack of targeting effect and spatiotemporal accuracy, as well as possible deleterious effects if the applied temperature is not applied properly, direct modulation using exogenous thermal stimulation instead is difficult to achieve and has rarely been reported. Therefore, the thermal responsive modulation methods currently used tend to be indirect methods by virtue of the transformation between other signals and heat, such as photothermal [34, 35], magneto-thermal [33, 42], etc. Among all the indirect thermo-responsive approaches, photothermal techniques attract the main attention because NIR exhibits excellent photothermal effect and deep penetration, based on the large family of photothermal nanoparticles. And photothermal-responsive neuromodulation approaches, which generate thermal effect on cytomembranes to modulate thermal-sensitive ion channels by transforming exogenous light signals to heat by photothermal nanoparticles, have developed to be one of the most frequently researched subfields of light-responsive neuromodulation. Currently, the exploration of photothermal-responsive neuromodulation for regulating neural activities and treating neurological diseases is in full progress.

**Fig. 2:**
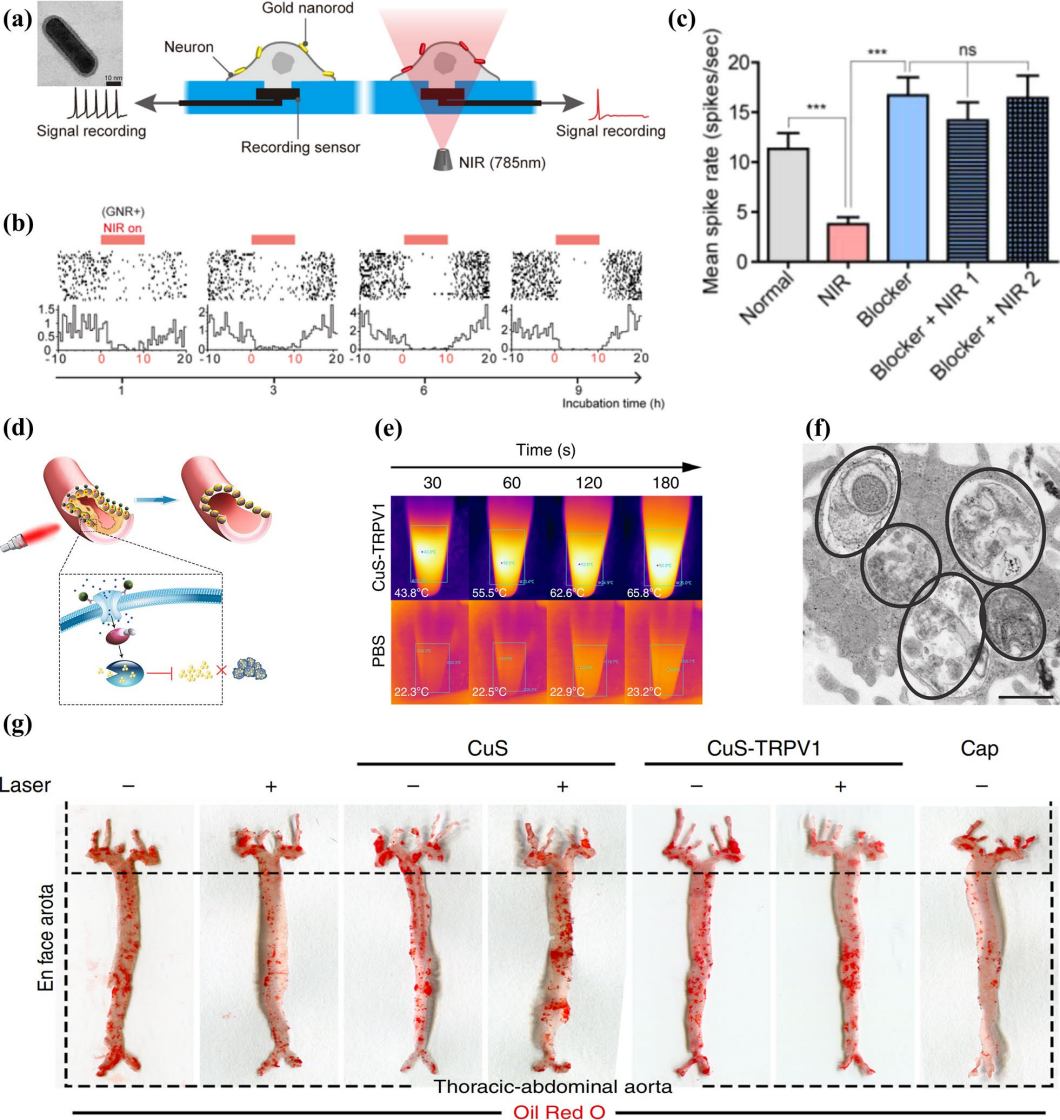
**a** Schematic diagram showing the photothermal-responsive neuromodulation mediated by gold nanorods (GNRs). The built-in figure is the TEM result of NH_2_-PEG-GNR. **b** Spike rates of normal neuronal activities irradiated to periodic NIR. **c** Mean spike rates of GNR + NIR-treated neurons when TREK-1 channels were blocked. Reprinted with permission [34]. Copyright 2014, American Chemical Society (ACS). **d** Mechanism illustration of CuS-TRPV1 in NIR-triggered treatment of atherosclerosis. **e** Infrared thermal imaging of PBS without and with CuS-TRPV1 after NIR irradiation. **f** Typical TEM images (Scale bar: 1 μm) of autophagosomes which contains 3 independent wells taken from areas of vascular smooth muscle cells (VSMCs) which were treated by CuS-TRPV1 and exposed to NIR for thirty cycles, where the double-membrane of autophagosomes were emphasized by some circles in black. **g** Typical pictures of a face aortic sample (The aortic arch is in the top part of the dotted box while the thoracic-abdominal aorta is in the lower one). stained by Oil Red O to indicate the lesion area after the treatment of CuS, CuS-TRPV1 and capsaicin (Cap). Reprinted with permission [35]. Copyright 2018, Springer Nature

By targeting thermal-sensitive ion channels on nerve cells, photothermal-responsive neuromodulation could instantaneously or continuously control neuronal activities. Yoo et al. [34] attached gold nanorods (GNRs) to nerve cell cytomembranes to achieve temporary inhibition or continuous modulation of them to NIR to generate photothermy (Fig. 2a). They evaluated the modulating effect of GNRs on natural nerve activities and it was proved that lower levels of activities were observed in GNR-pretreated nerves which were exposed to NIR, followed by recoveries to the original states when removing the NIR irradiation, indicating the potential of this method to precisely and reversibly modulate nerve actions (Fig. 2b). The modulating mechanism was also explored by blocking TREK-1 channels using fluoxetine, resulting in the disappearance of inhibitory effects on neuronal activities, which provided a strong argument for the tight link of this ion channel to GNR-induced photothermal-responsive neuromodulation process (Fig. 2c). In conclusion, as a typical photothermal-responsive and the first inhibition-based NNM method, this approach is promising in terms of reproducible and reversible modulation of neuronal activity and long-term control without cell damage.

Beside of controlling neural activities, photothermal-responsive neuromodulation also holds great promise for the treatment of neurological diseases. For example, Wen et al*.* constructed a switch for photothermal activation of the TRPV1 signaling pathway by combining copper sulfide (CuS) nanoparticles with TRPV1 monoclonal antibodies (CuS-TRPV1 NPs) to alleviate atherosclerosis through nanoparticle-mediated photothermal modulation of TRPV1 [35]. Upon exposing NIR irradiation, the thermo-sensitive TRPV1 cation channels in VSMCs were activated to allow a Ca^2+^ influx. In oxidized low-density lipoprotein (oxLDL)-treated VSMC, increased Ca^2+^ signal influx will induce autophagy and further impede foam cell (the source of atherosclerosis) formation (Fig. 2d). The CuS-TRPV1 NPs irradiated by 980 nm of NIR laser presented a rapid temperature elevation from 19.9 °C to 65.8 °C in 3 min (Fig. 2e), introducing a promising remote activation of TRPV1 channels by CuS-TRPV1-based photothermal switch. Then CuS-TRPV1-triggered photothermal switch was used in treating oxLDL-treated VSMCs under the irradiation of NIR laser to investigate its influence on the autophagy process and lipid accumulation in vitro. It could be found that with the presence of CuS-TRPV1 and NIR treatment, oxLDL-treated VSMCs exhibit significant double-membrane structural characteristic of autophagosomes under TEM observation, manifesting an effective autophagy, which will inhibit the formation of foam cell (Fig. 2f). The in vivo anti-atherosclerotic efficacy of CuS-TRPV1 was further evaluated by comparing the reduction of lesion area in different groups of en face aorta of plaque-bearing ApoE^−/−^ mice. It turned out that the CuS-TRPV1 + NIR treatment was not only the most effective (compared to the other controls) but also the only targeted treatment (it also reduced lesion area in the thoracoabdominal aorta compared to Cap), demonstrating the superiority of photothermal-targeted limitation of atherosclerotic progression (Fig. 2g).

### Photoconversion-responsive neuromodulation

Light, as one of the easiest ways to supply energy, can be physically transformed to achieve wavelength changes, which has unparalleled advantages for neuromodulation. Therefore, upconversion nanoparticles (UCNPs)-assisted neuromodulation of light response has attracted high scientific interest. In general, there will always be longer wavelengths in the emitted spectrum comparing to the input one during the photoluminescent process, according to the Stokes law. And the processes violating this law to emit spectra with shorter wavelengths are named as upconversion luminescence (UCL), in which varieties of sequentially absorbed lower-energy photons will be converted into higher-energy ones [50, 56, 57]. As the core tool of UCL, UCNPs have emerged a range of materials with various compositions, structures as well as absorption and release spectra. Among all types of UCNPs, the ones doped with rare earth elements perform tremendous potential in neuromodulation due to their typical ability to be excited by NIR to emit optical or UV light. Because of its penetration depth and little damage to biological tissues, NIR light is the most commonly used exogenous stimulus signal in light-responsive neuromodulation, while optical or UV light can modulate various neural activities in the human body [56].

**Fig. 3:**
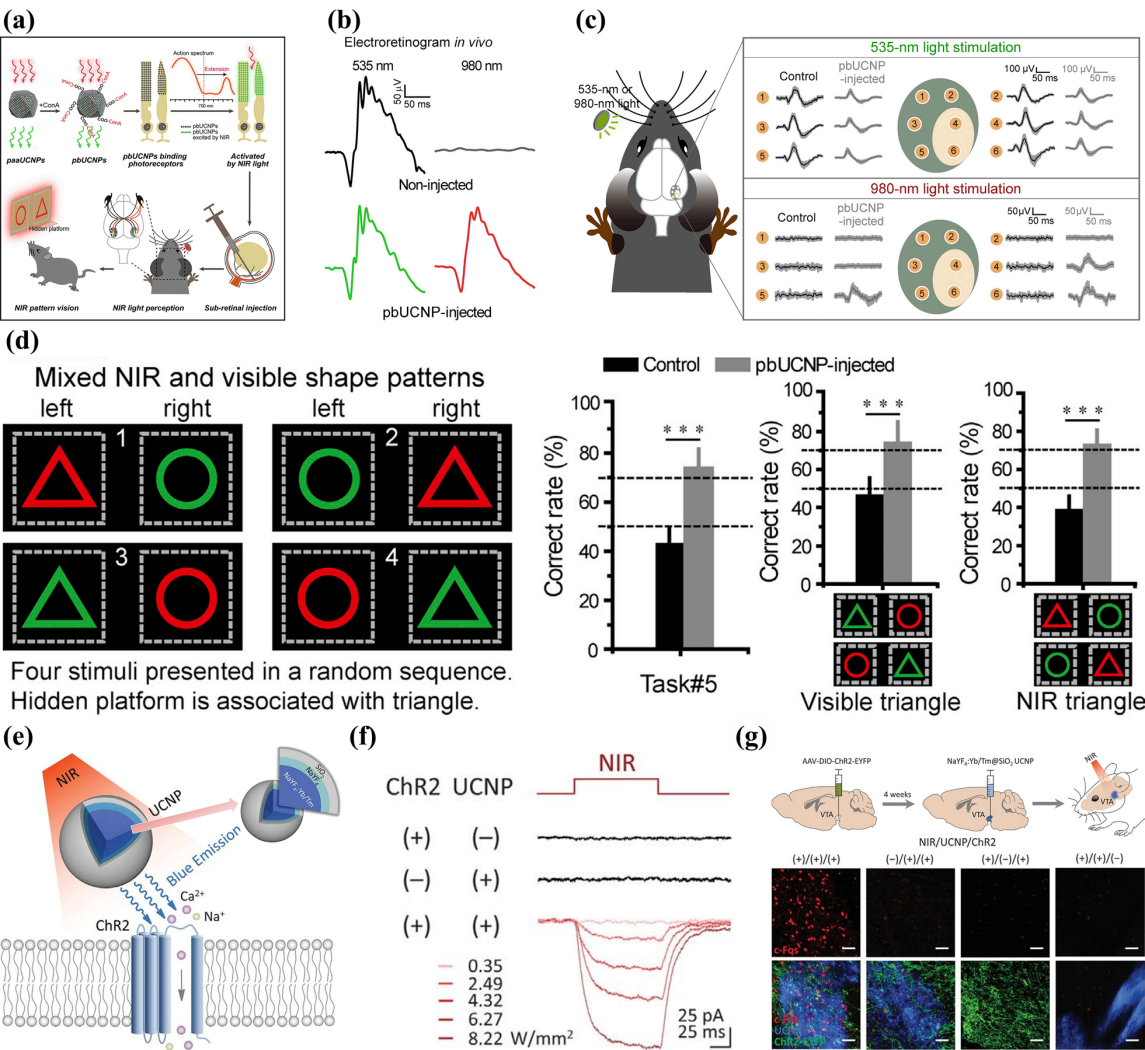
**a** Flow diagram showing the development of NIR vision in mouse models by injecting self-powered nanoantennae into sub-retinal areas. **b** Electroretinograms (ERGs) of mice irradiated to visual and NIR spectra. **c** Schematic illustration and results of the visual-evoked potential (VEP) captured from several locations in untreated and pbUCNPs-injected mice’ visual cortices irradiated to visual and NIR spectra. Location 4 and 6 situated in the binocular area while the other four belong to the monocular area. **d** The percentage of mice to simultaneously make proper discrimination among shapes emitting two types of spectra. Reprinted with permission [40]. Copyright 2019, Elsevier. **e** Schematic diagram showing optogenetics mediated by UCNPs + NIR. The inset shows the core–shell structure of the NaYF_4_:Yb/Tm@SiO_2_ particle emitting blue light. **f** Photocurrent generation condition of Ventral Tegmental Area (VTA) under the irradiation of NIR for 100 ms. **g** Experimental schematic diagram and confocal images showing the expression condition of *c-Fos* in mice’ VTA. Reprinted with permission [39] Copyright 2018, American Association for the Advancement of Science (AAAS)

Light-light conversion has the most intuitive application in the modulation of the visual nervous system. For example, Ma et al. [40] injected the photoreceptor-binding UCNPs (pbUCNPs) into the subretinal region to absorb NIR and then emit light for side-effect-free NIR vision sense (Fig. 3a). Electroretinogram (ERG) results showed that mice injected with pbUCNPs performed similar responsive curves when irradiated to optical spectrum and NIR, on the contrary of the ones that were not injected, demonstrating the ability of pbUCNPs with NIR to activate cone photoreceptors (Fig. 3b). When light at a wavelength of 535 nm was applied to six sites in the visual cortex of mice, the activation behavior indicated the perception of images, and visual evoked potentials (VEPs) were observed at each site of the control group as well as at the sites injected with pbUCNPs. However, such potentials were only performed on pbUCNPs injection sites of the latter when irradiated to NIR (980 nm), suggesting that pbUCNPs injection endowed mice with visual perception ability of near infrared light (Fig. 3c). Furthermore, the ability of NIR pattern vision was evaluated using previously trained mice which would only select triangle signboards if they could see. They presented pairs of lamps with randomly combined shapes (circle or triangle), orientations (left or right) and light types (visible or NIR). It turned out that only the ones with pbUCNPs-injection successfully chose the triangle lamps regardless of the other two characteristics, which indicated that this nanoantennae offered mice ability to visually sense optical as well as NIR spectra at the same time, both of which were utilized in the regulation process of their behaviors (Fig. 3d). Therefore, this typical upconversion-assisted light-light responsive neuromodulation technique could serve as a practical approach for extending the visible spectrum of mammals to NIR region, providing guidance for subsequent studies to facilitate the application of this field in civilian or military applications. It also suggested that non-genetic NNM technologies not only play roles in therapeutic purposes, but also provide potentials for enhancing our sensing abilities beyond the physical limitations.

Photo-responsive neuromodulation based on UCNPs has also facilitated optogenetics research. UCNPs-assisted optogenetics refer to a novel method of converting optical or UV spectra from NIR by lanthanide-based UCNPs to achieve indirect activation of typical optogenetic proteins with excellent spatio-temporal precise and minimal invasiveness [52, 53]. In order to overcome the limitation that visible wavelengths are difficult to penetrate deep into the brain, Chen et al*.* [39] applied UCNPs doped with lanthanide to transform tissue-penetrating NIR into higher-energy visible spectrum enough to activate deep-brain neurons expressing channelrhodopsin, resulting in transcranial optogenetics stimulated by NIR (Fig. 3e). They doped Yb^3+^/Tm^3+^ into NaYF_4_ nanocrystals emitting blue light and decorated them with SiO_2_ shells to synthesize NaYF_4_:Yb/Tm@SiO_2_ UCNPs with uniform pore sizes (Fig. 3e), achieving activation of the photosensitive channelrhodopsin-2 (ChR2) and verifying that the UCNPs are biocompatible. Then they evaluated the in vitro modulation result on a deep-brain area VTA of mice with dopamine (DA) neurons which expresses ChR2. After NaYF_4_:Yb/Tm@SiO_2_ injection and NIR irradiation, photoelectric currents were generated in ChR2 of VTA DA neuron and their intensities were improved following the elevation of intensities of applied NIR (Fig. 3f). Furthermore, the in vivo stimulating effect was estimated via the expressing level of *c-Fos*. Excitation only appeared in the NIR + ChR2 + UCNP group, which was proved by the significantly enhanced *c-Fos* expression levels. This result indicated that the excitation of postsynaptic constructions in target neurons was successfully triggered, which proves the prospect of in vivo application value of this method (Fig. 3g). In summary, UCNPs-assisted optogenetics, an almost noninvasive method with flexibility and robustness assisted by nanotechnology, enables minimally invasive regulation as well as long-distance treatment on optical neurons.

### Photochemical-catalytic responsive neuromodulation

As one of the most innovative prototype of non-genetic NNM, light signal not only modulates neural activity based on the activation or inhibition of ion channels through physical change, but also expands the mechanism of neuromodulation by taking advantage of the catalysis function of some light spectra towards the reactions of some functional molecules like neurotransmitters and drugs [38]. This newly expanded subareas was defined as photochemical-catalytic responsive neuromodulation. It relies mainly on the catalytic production of neurotransmitters, thus extending the regulatory target from the production of signals within a nerve cell to the transmission of signals between nerve cells, embodying the infinite possibilities of non-genetic NNM.

**Fig. 4:**
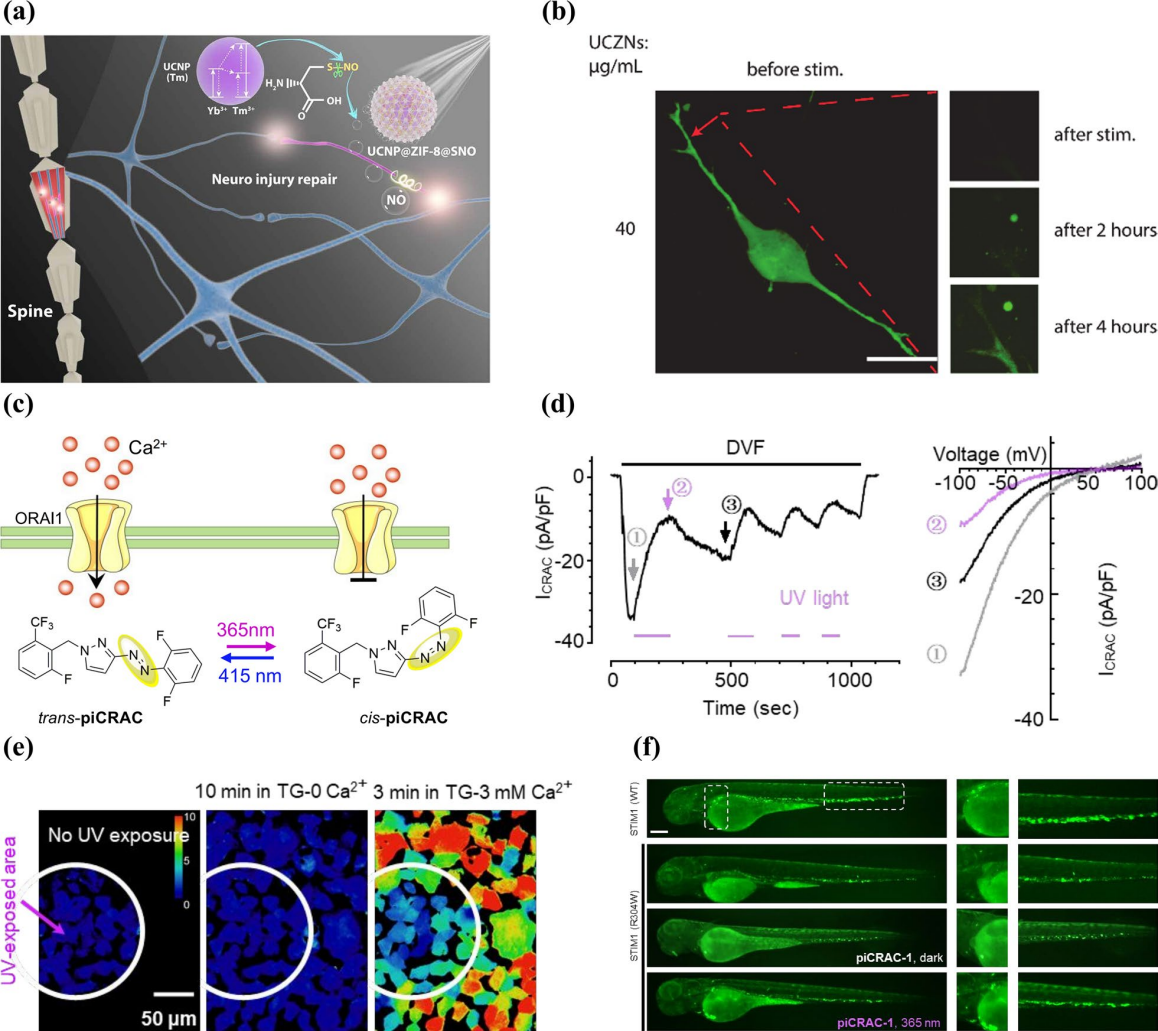
**a** Schematic illustration of traumatic SCI repair based on NIR-triggered NO release. **b** Morphological changes of UCZNs-treated dorsal root ganglion (DRG) neurons upon 980 nm light stimulation. The red arrows mark the damage region caused by highly treating power density of NIR (Scale bar, 25 μm). Reprinted with permission [38]. Copyright 2020, AAAS. **c** Schematic diagram showing the molecular structure, light-induced isomerization and the gate effect on ORAI1 channel of piCRAC-1. **d** Representative whole-cell currents (left) and I-V relationships of three time points (right) of ORAI1-SS-GFP-expressing HEK293 cells in DVF solution having piCRAC-1. **e** Representative areas of HEK293 cells performing piCRAC-1-induced local modulation on store-operated calcium entry (SOCE). **f** Lateral images showing Tg (CD41: EGFP) embryos which express R304W activating mutant or STIM1 in wild type (WT) with or without piCRAC-1. Reprinted with permission [58]. Copyright 2020, ACS

Nitric oxide (NO) features a key vertebrate biological messenger, serving as a promoter in neural growth and repair, especially in profiting accident-caused nerve injury diseases like traumatic spinal cord injury (SCI) [59, 60]. To achieve on-demand NO release at the lesion region of traumatic SCI, Jiang et al. [38] reported a UCNP-engineered zeolitic imidazolate framework-8 (ZIF-8) with nitrosothiol (CysNO) loading and named this nanocomposite as UCZNs. Upon exposing to NIR, UCNP core of UCZNs converts NIR input to UV output, which further breaks the S-NO bond of CysNO for NO generation (Fig. 4a). To quantify the in vitro neuron development and outgrowth activities, DRG neurons derived from Sprague–Dawley rats were treated by UCZNs (40 μg ml^−1^) and NIR light. Upon 980 nm laser exposure, the previously damaged regions (especially terminals) of the axons regenerated in 4 h, which was indicated by the enhanced neuron calcein signal, offering the hope for in vivo SCI treatment (Fig. 4b).

In addition to neurotransmitters release, the application of photochemical catalysis to generate ion channel modulators to remotely and controllably activate or inhibit ion channels is also an important component of photochemical-catalytic responsive neuromodulation. Ca^2+^ release-activated calcium (CRAC) [59] channels modulates a lot of calcium-regulated reactions in vivo and their overactivation may lead to a number of diseases, such as Stormorken syndrome [61]. Using bioisosteric substitution in store-operated calcium entry (SOCE) inhibitors derived from heterocyclic N-aryl benzamide, Yang et al. [58] designed a group of photo-switchable regulators of CRAC channels and termed them piCRACs, in which the azoster moiety enabled reversible photoconversion between their cis and trans isomerides, thus allowing them to inhibit CRAC (Fig. 4c). The result of whole-cell currents showed that piCRAC-1 reduced *I*_*CRAC*_ in HEK293 cells when irradiated to UV and the inhibition could be temporally reversed, and only the areas with UV irradiation performed obvious cell SOCE decline, which together demonstrated the spatio-temporal inhibition ability of piCRAC-1 on cell SOCE (Fig. 4d, e). Further research in zebrafish embryos with Stormorken syndrome proved that with UV (365 nm) irradiation, piCRAC-1 led to significant enhancement in maturing thrombocytes to rescue thrombocytopenia, indicating that piCRAC-1 would be able to achieve photo-controllable modulation of CRAC and cure diseases related to its overactivity (Fig. 4f). In conclusion, this photo-pharmacological spatio-temporal inhibition method could promote research on the relationships between the structures and functions of calcium channels, the interrogation of human calcium-regulated signals, as well as diseases connected with disordered calcium activities.

### Photoelectric-responsive neuromodulation

Although the traditional direct electrical neuromodulation based on large medical devices has some shortcomings such as poor spatial accuracy and the risk of infection, electrical signals as a direct manifestation of neural excitation have not been ignored in the field of NNM. Through the mediation of nanoparticles, the conversion of other physical fields to electrical signals is conveniently achieved, thus giving birth to remote non-invasive and accurate indirect electrical-responsive neuromodulation. Among them, due to the high penetration and spatiotemporal accuracy of NIR and the existence of well-researched photoelectric nanomaterials, photoelectric-responsive approach has become the most rapidly developing indirect electrical stimulation neuromodulation and an important branch of light-responsive neuromodulation.

**Fig. 5:**
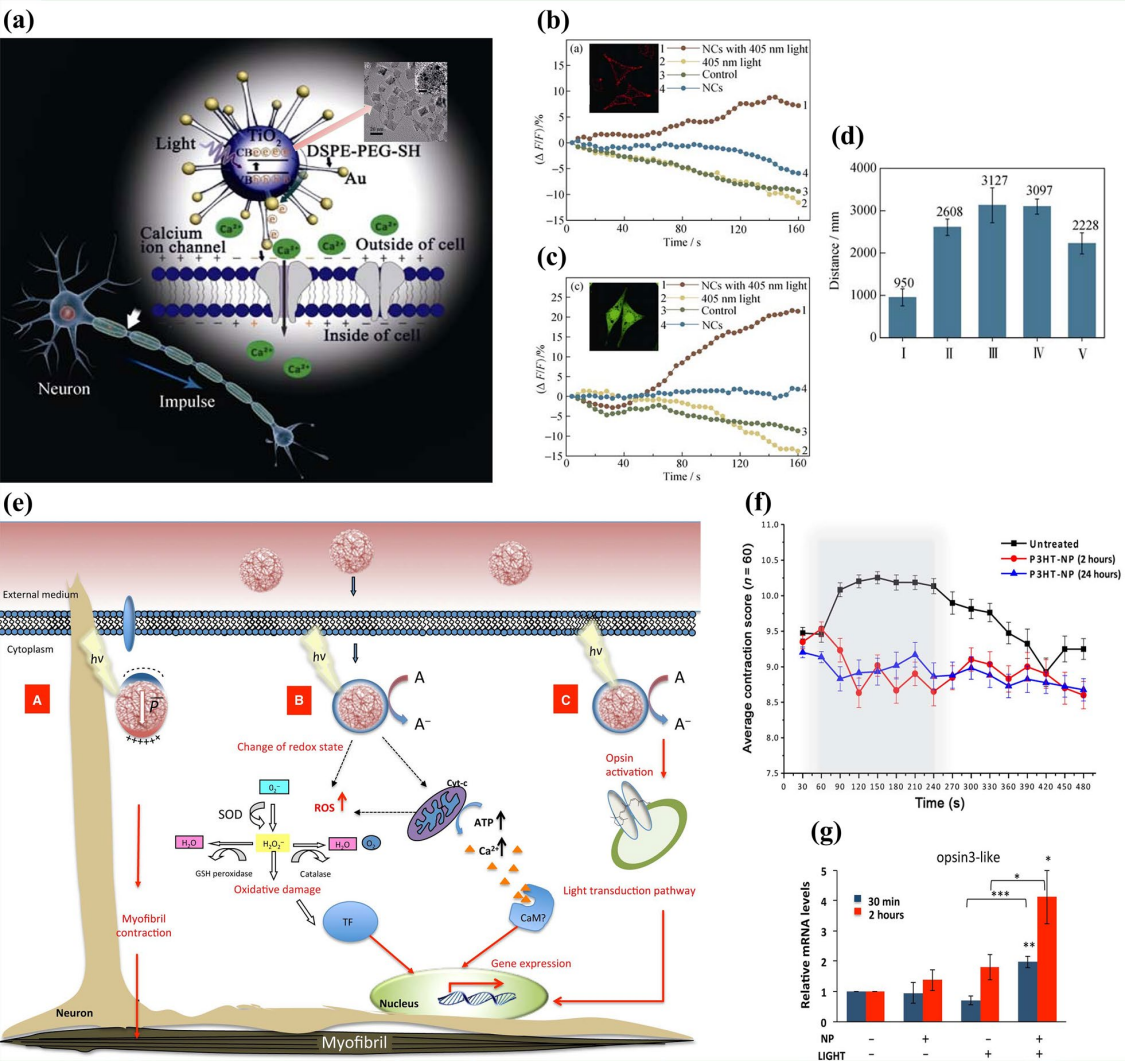
**a** Schematic diagram showing the depolarization of nerve cells triggered by 405 nm laser-irradiated PENCs. Inset is the TEM result of PENCs. Fluorescence signal changes of **b** Di-8-ANEPPS and **c** Cal-520 of PC12 cells. **d** Moving distance at speeds faster than 20 mm/s of five groups of zebrafishes including (I) control, (II) epileptic, (III) PENCs, (IV) laser and (V) PENCs + laser. Reprinted with permission [62]. Copyright 2017, Science Press. **e** Schematic illustration of in vivo reactions triggered by light-irradiated P3HT-NP. **f** The P3HT-NPs-induced responding behaviors of *H. vulgaris* in one cycle of light. **g** P3HT-NPs’ photoactivation effect on the expression of opsin3-like gene. Reprinted with permission [23]. Copyright 2017, AAAS

Photosensitive nanomaterials with semiconductor properties can generate electrical current if they are exposed to light with energy higher than the band gap energy of them. Jiang et al. [62] designed the photoelectric nanocomposites (PENCs) by bonding gold nanoparticles on the surface of nanoscale photosensitive TiO_2_ semiconductors (Fig. 5a). They stained different groups of PC12 cells respectively with Di-8-ANEPPS (instructing cytomembrane potential variation) and Cal-520 (indicating Ca^2+^ influx), and it turned out that only PENCs + laser group performed remarkable fluorescence intensity enhancement of both the two dyes, which strongly demonstrates that the photo-triggered electrical signal generated by PENCs results in cell membrane depolarization and Ca^2+^ influx (Fig. 5b, c). The same results were obtained from the in vivo research on zebrafishes with epilepsy, where only PENCs + laser treated ones performed significantly less seizure or faster movements, which are the main symptoms of epilepsy (Fig. 5d). In conclusion, this photoelectric neuromodulation approach assisted by PENCs has great significance for the treatment of neurological diseases.

Similar to photoconversion-responsive neuromodulation, photoelectric-responsive approaches could also be applied to optogenetics. Tortiglione et al. [23] achieved photo-triggered regulation on behaviors of eyeless fresh water polyp Hydra vulgaris (*H. vulgaris*) using a poly(3-hexylthiophene) nanoparticle (P3HT-NP). When exposed to the optical light, P3HT-NP will generate electrons that lead to a variety of biological responses, including (A) influencing local neurons to modulate contracting behaviors, (B) enhancing the expression of targeted genes or (C) initiating light-dependent molecular cascades reactions (Fig. 5e). The influence on behaviors was measured on polyps, whose contracting behaviors were observed in an 8 min cycle of light and evaluated using a points-scoring standard where 6 means ultimately contracting and 11 means extremely elongating. The polyps are all relaxed within the first minute of ambient light, and the P3HT-NP-treated polyps exhibited a tendency to suppress or even contact elongation during the subsequent 3 min white light-emitting diode (LED) period, compared to the normal elongation behavior of the control group. Moreover, the P3HT-NP-treated samples continued to contract, while the samples in the control group obviously recovered normal elongating/contracting behavioral periods during the last 4 min of ambient light (Fig. 5f). Then the influence of P3HT-NP on light-responsive opsin3-like gene was analyzed using quantitative reverse transcription polymerase chain reaction (qRT-PCR) after the irradiation of LED. And opsin3-like showed significantly higher expression level (Fig. 5g). These behavioral and genetic studies conformed that P3HT-NP not only improves the contraction of the polyps, but also modifies the transcription of opsin3-like genes. Hence, P3HT-NPs serve as light nanotransducers to enhance photosensitivity in animals, offering the possibility of using light to modulate behaviors and providing new ideas for the biomedical field.

### Photomechanical-responsive neuromodulation

There are abundant mechanical-responsive ion channels distributed on the nerve cell membrane, such as Piezo1, Piezo2 [24]. However, the severe lack of spatial precision of the directly applied mechanical force has prevented the application of such mechanical signals for direct stimulation of nerve cells in the field of neuromodulation for a long time. Nowadays, with the development of nanosynthesis and functionalization technologies, it is possible to convert other signals into mechanical force at the accuracy of single cell or even molecular level, and photomechanical conversion is one of the important ways, which provided a new approach to regulate activities of a variety of cells like nerve cells, blood platelets as well as T cells. Specifically, to nerve cells, novel optomechanical nanoparticles can convert NIR with strong penetration power and high spatial–temporal resolution into mechanical signals that can directly stimulate nerve cells, giving rise to photomechanical-responsive neuromodulation, which is another important branch of photo-responsive neuromodulation.

**Fig. 6:**
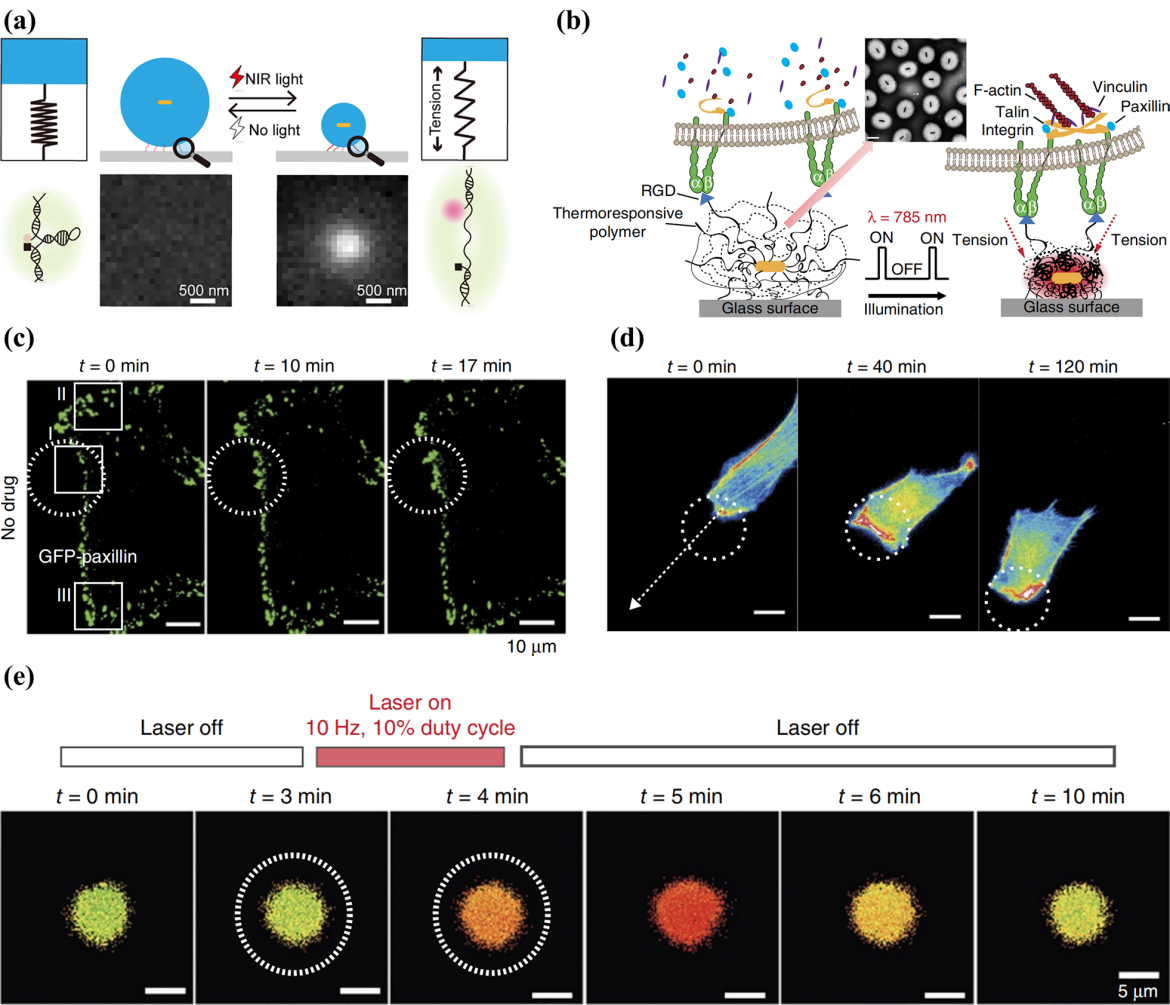
**a** Schematic diagram showing Polymer Force Clamp (PFC) assisted force spectroscopy at the level of single biological molecules. Reprinted with permission [63]. Copyright 2018, ACS. **b** Schematic illustration of optomechanical actuator (OMA), its construction and operation mechanism. Inset is the TEM result of OMA. **c** Time-lapse total internal reflection fluorescence (TIRF) results of GFP-paxillin-transfected NIH/3T3 cells which were cultivated on OMA. NIR irradiation areas were inside the white dotted circles. Green spots are RGD peptide inside the cells. **d** Time-lapse results of the migration behaviors of NIH/3T3 cells stimulated by OMA. **e** Variation of the fluorescence intensity of Ca^2+^ indicator Fura-2 in OMA + NIR-treated T cells. Reprinted with permission [64]. Copyright 2016, Springer Nature

Early attempts of nanoparticle-induced opto-mechanical conversion were first applied to force spectroscopy to investigate the mechanical expansion behavior of biological molecules under mechanical force. Su et al. [63] designed a core–shell optomechanical nanoparticle consisting of a photothermal plasmonic nanorod coated by thermo-sensitive polymer. When irradiated by NIR, photothermal effect of nanorod core results in rapid temperature increase to collapse the polymer shell, where NIR was transformed to mechanical force to efficiently and controllably unfold interested molecules (Fig. 6a).

Subsequently, the nanoparticle-based optomechanical actuator (OMA) reported by Liu et al*.* [64] introduced nanoparticle-induced photomechanical conversion to the manipulation of living cell behaviors, laying the foundation for its further application in neuromodulation. When irradiated by NIR, instantly shrink will occur on OMAs, generating mechanical force on receptor-ligand complexes connecting the fixed particles and targeting cell membrane (Fig. 6b). To explore the effect of OMA on cell behaviors, they transfected GFP-paxillin (surrogate marker of FAs) to NIH/3T3 cells and cultured them on one layer of OMA. In NIR-irradiated region, FA size performed obvious growth (Fig. 6c), suggesting that OMA-induced opto-mechanical conversion can recruit key molecules for cell survival like GFP-paxillin. And prolonged stimulation of cells by LifeAct-mCherry promoted the formation of protrusions and subsequent movement of the whole cell (Fig. 6d), demonstrating that NIR + OMA stimulation has the potential to control cell migration. Finally, they applied opto-mechanical stimulation to receptors of T cells (TCRs) through NIR + OMA, and T cells performed instant Ca^2+^ influx (Fig. 6e), the indicator of activation behavior, showing the possibility of OMA to optically modulate the activation of T cells. Taken together, photomechanical approaches show great promise in remote and precise control of molecule movement, cell displacement and cell activation, and will be another branch of light-responsive neuromodulation worth exploring.

In summary, based on the diverse and growing types of application scenarios, photo-responsive neuromodulation will long remain as the most promising subfield of non-genetic NNM, offering hope for the treatment of increasing numbers of neuro-related diseases through the design and collocation of different signal types, nanoparticles, and action sites.

## Magnetic-responsive neuromodulation

Among the various physical stimuli which have been employed to target neuromodulation, magnetism highlights prominent merits including remote interventions and deep penetrations towards biological subjects [33, 41]. The establishment of remote neuromodulation systems through magnetic response not only benefited from its deep penetration in living bodies, but also profited by its weak interaction with biomolecules. Such miniaturized, remote, and minimally invasive strategy for neuromodulation has practical implications in deep brain treatment and has become the core competitiveness that distinguishes magnetic-responsive neuromodulation from other branches. Although direct stimulation of magnetically sensitive ion channels has not been reported yet, the rapid development of functionalized nanoparticles has realized the transformation of magnetic signals to other physical fields and even the influence of magnetic field on the local microenvironment of nerve cells, making magnetic-responsive neuromodulation the second major branch of NNM after photo-responsive strategy. The current NNM strategies induced by functionalized magnetic nanoparticles mainly include magneto-thermal, magneto-mechanical, magnetoelectrical and magnetically induced microenvironmental responsive approaches, each of which have led to a novel mechanical neuromodulation strategy [65].

### Magnetothermal responsive neuromodulation

**Fig. 7:**
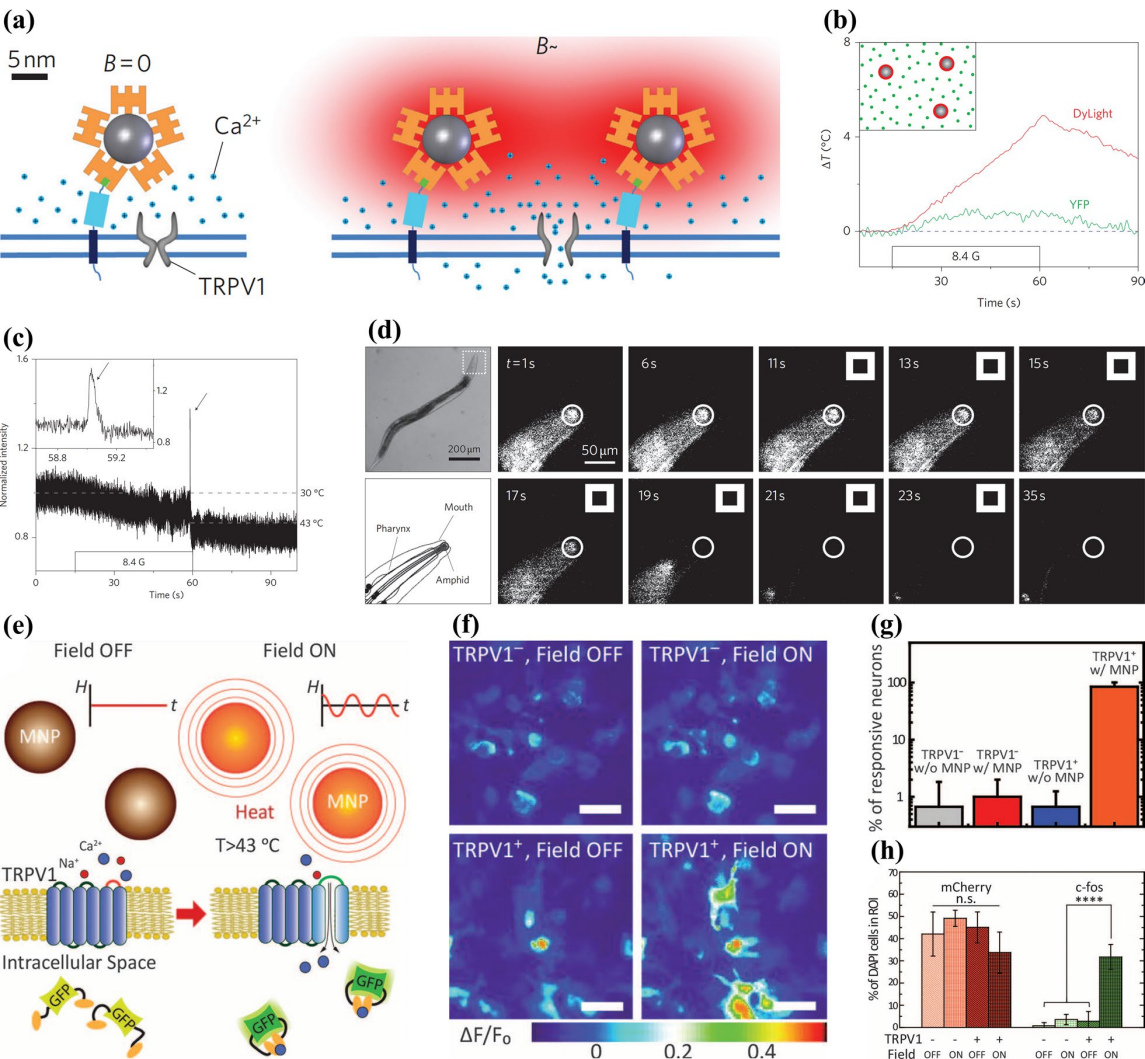
**a** Schematic illustration of the heat (red)-triggered opening of TRPV1 in a RF magnetic field (B) achieved by the local temperature rise of superparamagnetic MnFe_2_O_4_ nanoparticles (grey) which are coated by streptavidin-DyLight549 (orange) and connected on the cytomembrane by the AP (green box)-CFP (cyan box)-TM (blue box) protein. **b** The comparison of a nanoparticle-triggered heating effect of the nanoparticle surface (red, DyLight549 fluorescence) to the solution (green, YFP fluorescence) when it was applied to a RF magnetic field. **c** The membrane potential of RF magnetic field-treated hippocampal neurons (expressing TRPV1 specifically) which are coated by nanoparticles. **d** Images of *C. elegans*’ thermal-avoiding reaction to the remote regulation indicated by the fluorescent intensity of the nanoparticles’ PEG coatings. Reprinted with permission [33]. Copyright 2010, Springer Nature. **e** Schematic diagram showing the magnetic field-induced temperature increase of magnetic nanoparticles (MNPs) to stimulate TRPV1. **f** Fluorescence images of differently treated HEK293FT cells. **g** The number of nerve cells which performed spike in no more than five seconds after magnetic treatment. **h** Percentage of nerve cells performing mCherry and *c-Fos* in four differently treated groups. Reprinted with permission [68]. Copyright 2015, AAAS

Widely reported superparamagnetic nanomaterials could conveniently achieve transformation from magnetic signal to thermal effect [66]. Similar to photothermal responsive neuromodulation, such an advantage make it possible to regulate heat-sensitive ion channels on the surface of nerve cells by external magnetic signals. The combination of advantages in materials, ion channel pathways, and signal transduction make magnetothermal responsive approach the most developed subarea of magnetic-responsive neuromodulation. Huang et al*.* [33] have achieved remote control of TRPV1, a typical temperature-sensitive ion channel, by the superparamagnetic nanoparticle of manganese ferrite (MnFe_2_O_4_) which can transform magnetic signal into local temperature elevation on TRPV1-expressing cells. Superparamagnetic nanoparticles coated with streptavidin-DyLight549 were anchored on the membrane by the bonding of AP-CFP-TM proteins and performs local heating in a radio-frequency (RF) magnetic field to turn on the thermal activated TRPV1 (Fig. 7a). Under RF magnetic field treatment, there was a significant difference in the temperature distribution between the nanoparticles and the dispersion. The former showed a high heating rate of 0.31 ℃ s^−1^, while almost no temperature change was observed in the latter, proving the effectiveness and targeting of such nanoparticle-mediated heating ability (Fig. 7b). The membrane potential of nanoparticle-anchored hippocampal neurons (specifically expressing TRPV1) was monitored by typical voltage-sensitive dye ANNINE6 in a RF magnetic field. As the cytomembrane temperature increased up to 40 ℃, an action potential was recorded by the fluctuation of ANNINE6 fluorescence, which proved that the thermal signal come from the nanoparticle was sufficient to elicit action potentials in the neurons (Fig. 7c). The further remote control of a behavioral response in live animals was performed on *C. elegans* model, which will intrinsically reflex and initiate backward locomotion under the instantaneous heating shock [67]. When put in the RF magnetic field (11–28 s), the fluorescent intensity of the anaesthetized *C. elegans’* head regions, which were treated by nanoparticles coated by PEG fluorescein, performed decrease as the temperature increased (20–34.8 ℃), reversed the moving directions of *C. elegans*, demonstrating the possibility of this approach in exploring neuronal activity and curing neuro-related diseases (Fig. 7d).

As an earlier developed direction, magnetothermal-responsive neuromodulation has already achieved wireless stimulation to deep brain areas. Chen et al. [68] applied magnetothermal Fe_3_O_4_ MNPs to noninvasively, reversibly and remotely excite TRPV1-expressing neurons with alternating magnetic field (AMF) (Fig. 7e). Using MNP-incubated HEK293FT cells, they firstly clarified the existence of magnetothermal-triggered calcium influx according to the intracellular calcium fluorescence images, where only TRPV1-expressing cells performed obvious fluorescence intensity increase during the magnetic stimulation (Fig. 7f). Similar results were found in TRPV1-expressing and MNP-cocultured primary hippocampal neurons, most of which could be evoked by a periodic magnetic field consisting of 10-s fields and 1-min intervals, further exemplifying the possibility of applying this magnetothermal strategy to human deep brain areas (Fig. 7g). They further designed in vivo experiment in VTA and quantified the excitation of VTA neurons by *c-Fos*, a gene whose expressing level is depended by cell activation degree. It was found that only magnetic field + MNPs groups performed obviously increased percentage of cells expressing *c-Fos*, demonstrating the neural activation of VTA (Fig. 7h). Such a remote and non-invasive deep brain stimulation technique will pose a challenge to traditional therapy in the area of treating serious neurological diseases. For example, the phasic excitation of VTA may be associated with the cure of major depression.

### Magneto-mechanical responsive neuromodulation

**Fig. 8:**
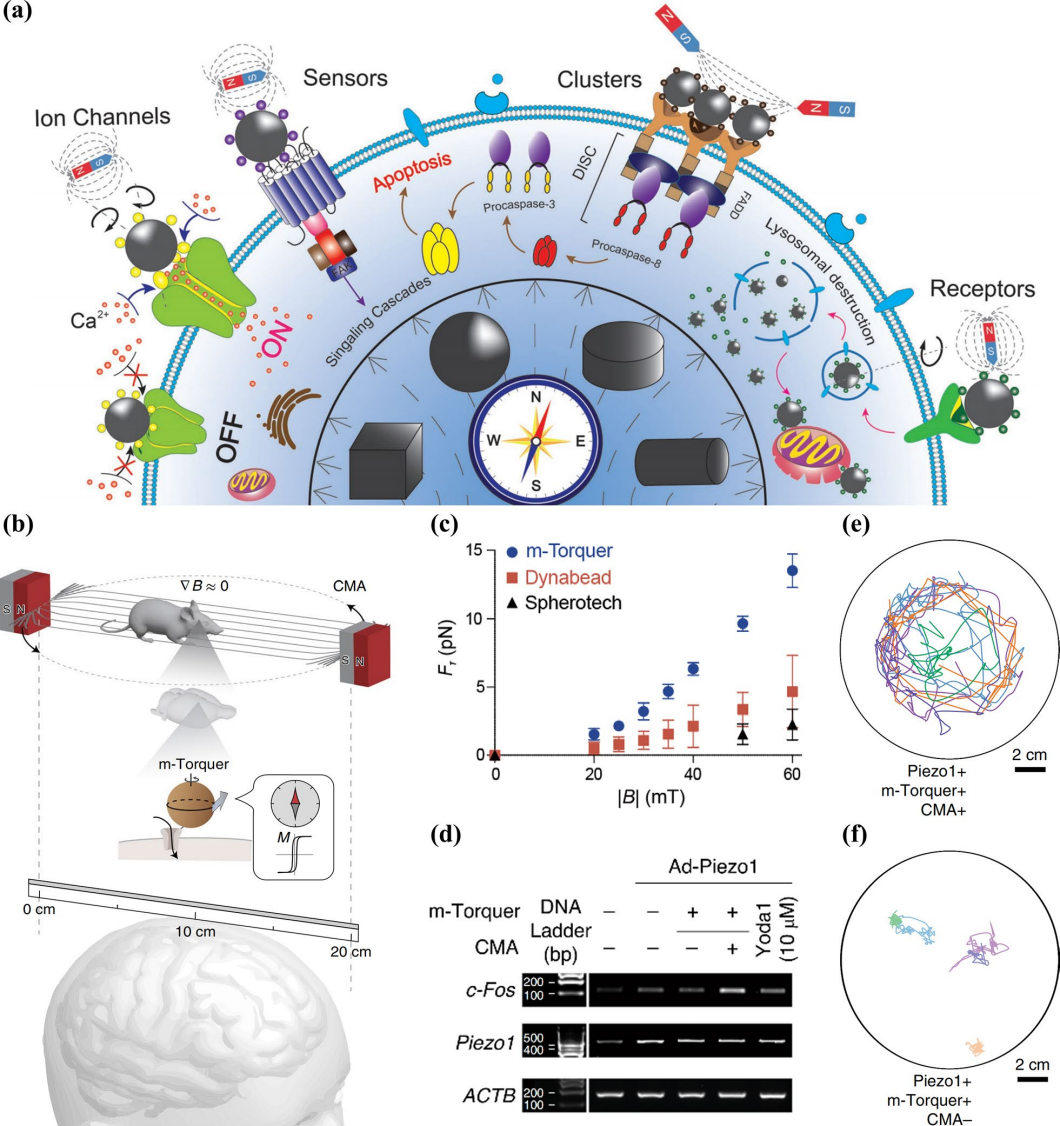
**a** Schematic diagram showing the transduction and switch processes of magneto-mechanical signal induced by magnetic nanomaterials for modulating the fate of cells. Reprinted with permission [69]. Copyright 2018, Wiley–VCH Verlag GmbH & Co. KGaA, Weinheim. **b** Structure diagram describing the m-Torquer nanosystem. **c** The comparison of generation ability of torque force in rotating CMA of m-Torquer to Dynabead and Spherotech, which are magnetic beads in the market. **d** The expression of *c-Fos* analyzed by PCR with reverse transcription (RT-PCR) in neurons which expressed Piezo1 and were stimulated by m-Torquer with a rotating CMA. β-Actin (ACTB) is a housekeeping gene and Yoda1 were used to activate Piezo1. The movement loca of 5 mice after being treated by m-Torquer with (**e**) and without (**f**) CMA. Reprinted with permission [41]. Copyright 2021, Springer Nature

Though optomechanical-responsive neuromodulation is still in the preliminary stage of exploration, magneto-mechanical responsive approaches are at the cutting edge of magnetic-responsive neuromodulation and represent an emerging potent prototype in the construction of mechanobiological systems, thanks to easier transformation from magnetic fields to mechanical signals. Indeed, magneto-mechanical nanoparticles had already played a role in biomedical field prior to the rise of NNM. For example, magneto-mechanical control of cells is a novel research direction, which includes (1) converting exogenous magnetic field into mechanical stretch to stimulate mechanical responsive ion channels and control ion influx; and (2) transmitting mechanical force through mechanosensors on cytomembrane to trigger signal cascades and biomolecule cluster construction to initiate apoptosis and organelle destruction [69] (Fig. 8a). All of the above laid the foundation for the application of magneto-mechanical nanoparticles to NNM.

Recently, magneto-mechanical responsive neuromodulation achieved by stimulating mechanosensitive ion channels has been reported. Lee et al. [41] constructed the nanoscale magnetic torquer (m-Torquer), which consists of a certain rotating uniform magnetic field derived from circular magnet array (CMA) and a m-Torquer generator that performs nanoscale magnetic compass to trigger torque force (*F*_*τ*_) for ion channel gating and conduct magnetomechanical force into biological targets. In the presence of m-Torquer, continuous and reproducible modulation of freely moving neurons in mice was achieved by stimulating Piezo1 channels that are sensitive to mechanical forces (Fig. 8b). Fluorescent probe along with Stokes’ law of viscous media were utilized to evaluate the *F*_*τ*_ produced by m-Torquer, which turned out to be 2–10 pN at a magnetic flux density of 20–50 mT (much higher than that of commercialized magnetic beads Dynabead and Spherotech), manifesting the capacity of providing enough interaction energy to generate mechanotransduction (Fig. 8c). Then they treated cultured mouse neurons from cortex with m-Torquer and put them into CMA. And the potential of m-Torquer to stimulate magnetomechanical neuromodulation was verified by the clear band representing *c-Fos* expression, the gene reporting calcium influx-activated neuron, in RT-PCR in comparison to the control groups (Fig. 8d). Furthermore, the mice’s freely moving behaviours in long distances were observed to analyze the neuromodulation of m-Torquer on the movement of mice. The tracking results demonstrated the mice showed a significant enhancement of motor function under m-Torquer stimulation with CMA (Fig. 8e, f).

### Magnetoelectrical responsive neuromodulation

**Fig. 9:**
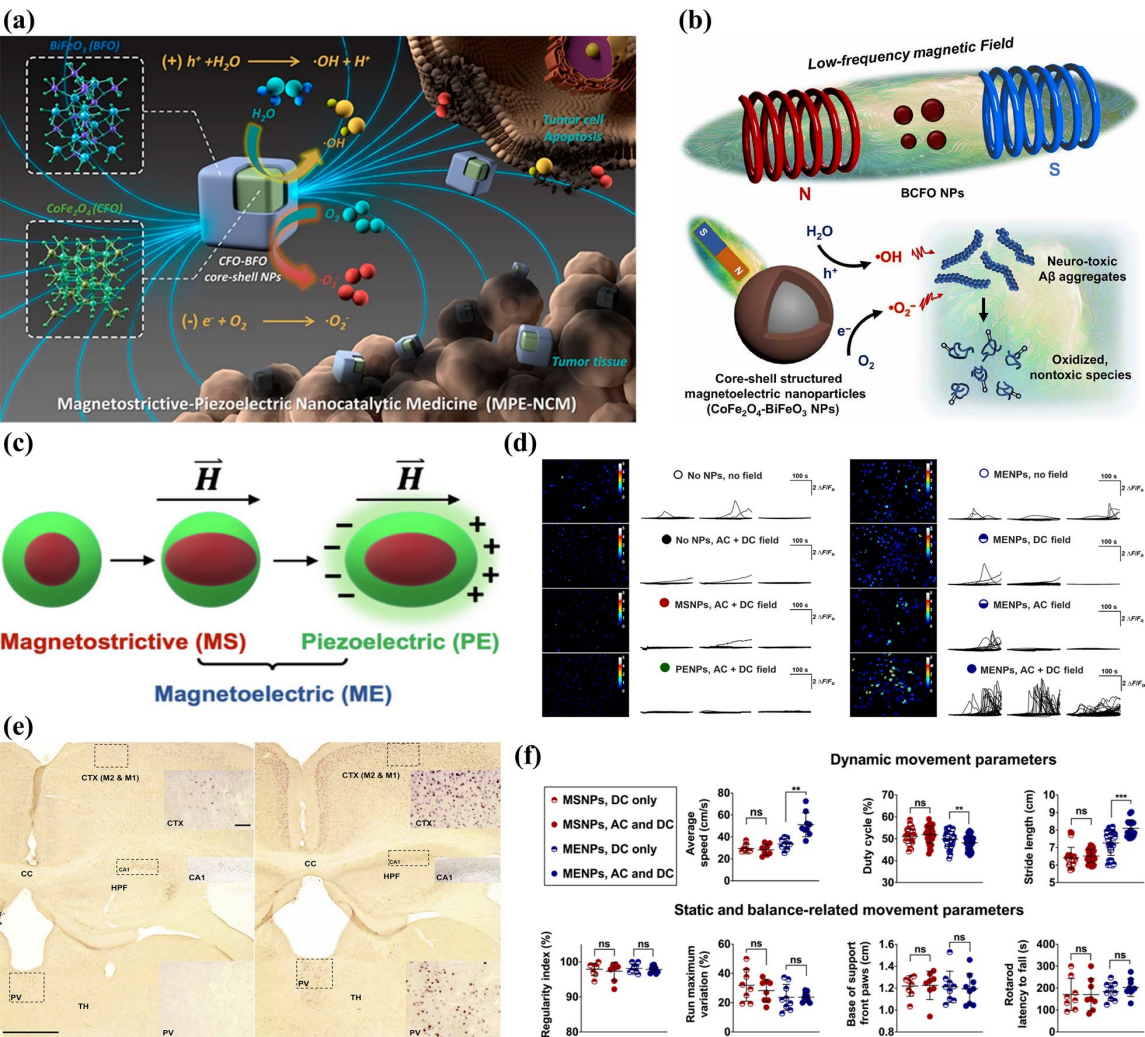
Schematic diagram of ROS production induced by CFO-BFO nanoparticles in AMF to **a** kill tumor cells and **b** dissociate Aβ aggregates of AD. Reprinted with permission [70]. Copyright 2021, ACS. Reprinted with permission [71]. Copyright 2022, AAAS **c** Schematic diagram of the double-phase material consists of magnetostrictive as well as piezoelectric components, where strain coupling effect plays a role to produce magnetoelectricity. **d** Calcium activities’ variations showing the effect of magnetic signals on MENPs to regulate neural activities in vitro. **e** MENP-induced increase of *c-Fos* expressing levels observed from motor cortex (CTX: M1 and M2) as well as limbic thalamus (PV). The external magnetic stimulating field is DC (left) and AC + DC (right), respectively. **f** Dynamic as well as static differences of differently-treated mice’ moving behaviors observed using a CatWalk device. Reprinted with permission [43]. Copyright 2021, AAAS

The conversion of magnetic field to electric signal could be achieved by the combination of magnetostriction and piezoelectric properties, and magnetostrictive-piezoelectric core–shell nanoparticles were previously used in the anti-tumor field to convert external magnetic fields into electrical signals. Ge et al. [70] constructed CoFe_2_O_4_-BiFeO_3_ (CFO-BFO) magnetostrictive-piezoelectric nanoparticle. Under the action of AMF, the magnetostrictive effect of CFO core will pull the BFO shell to deformation and occur piezoelectric effect, resulting in the separation of electrons and holes (Fig. 9a), which react respectively with water and oxygen dissolved in water to generate two reactive oxygen species •OH and •O_2_^−^ to kill cancer cells. Jang et al. [71] moved further to take advantage of the same nanomaterial to depolymerize the Aβ aggregates deposited in brain, a major pathological feature of AD, extending the application of magnetoelectrical nanoparticle to neurological disease treatment (Fig. 9b). Furthermore, it is reasonable to speculate that by adjusting the applied form and field strength of the magnetic field, the damage to nerve cells by ROS can be avoided on the basis of the generation of magnetically induced potential.

Unexpectedly, this idea of magneto-electrical conversion was also used in NNM. Through the strain coupling effect by magnetostrictive cobalt ferrite nanoparticle (MSNP) core with piezoelectric effect of barium titanate shell. Kozielski et al*.* [43] synthesized a double-phase magnetoelectric nanoparticle (MENP) as a nanoelectrode, which can trigger deformation inside cobalt ferrite in magnetic field to exert strain on the barium titanate, generating charge separations as electric modulating signals to achieve wireless neuromodulation (Fig. 9c). Alternating current (AC) along with direct current (DC) magnetic field caused obvious increase in the calcium-transient rate of MENPs-treated neurons compared to control groups, suggesting that MENPs in the magnetic field can wirelessly achieve neuromodulation in vitro (Fig. 9d). They also evaluated the expressing levels of *c-Fos* protein, which is commonly utilized to mark activities of cells, in several areas of mice’ brains, and detected significant increase in CTX only: M1 and M2 and PV of thalamus treated by MENPs along with AC + DC, which demonstrated that the nanoelectrodes were sufficient to achieve wireless deep brain stimulation (DBS) locally to modulate the basal ganglia circuitry (Fig. 9e). They further analyzed the gait statistics of mice in a CatWalk device and observed few variations in static or balance-related arguments between differently-treated groups, while parameters of dynamic movement performed significant changes in AC + DC and MENPs-treated mice, resulting in evidence that DBS triggered by nanoelectrodes and magnetic fields was sufficient to alter mouse behavior without inhibiting normal motor activity (Fig. 9f). This magnetoelectric method provided a versatile approach to implement neuromodulation techniques with less invasion and deeper brain-penetration, and holds great promise for related therapeutic applications.

### Magnetically induced microenvironmental-responsive neuromodulation

**Fig. 10:**
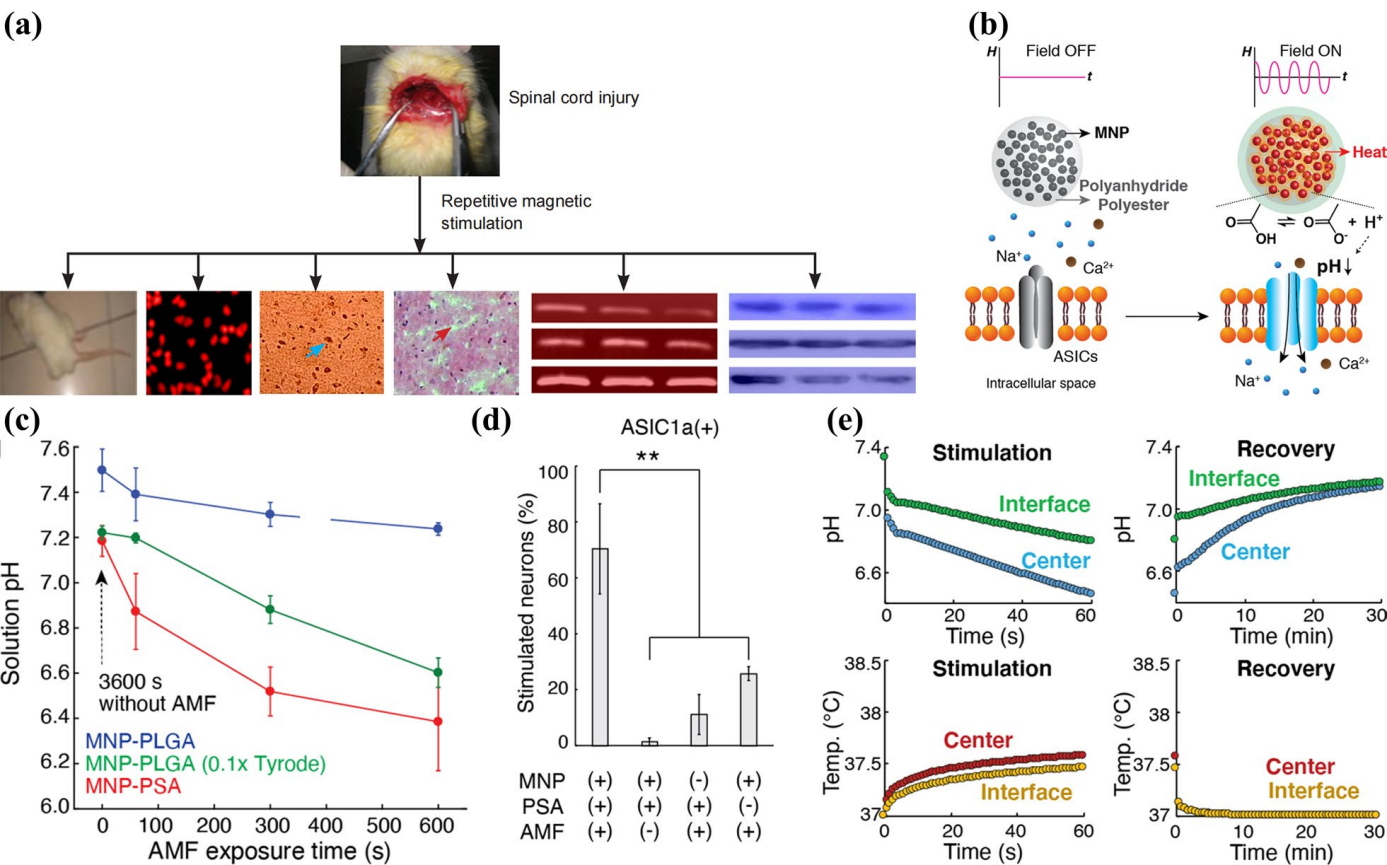
**a** Summary of magnetic-induced neuron protection and spinal cord repair. Reprinted with permission [72]. Copyright 2016, Wolters Kluwer Medknow Publications. **b** Schematic diagram showing the remote control of proton generation. **c** The stimulation proportions of differently-treated ASIC1a^+^ neurons. **d** pH variations of MNP-PLGA and MNP-PSA loading different solutions stimulated by AMF. **e** Variations of pH and temperature at the center and interface of injected MNP-PSA stimulated by AMFs in buffer systems simulating human cerebrospinal fluid. Reprinted with permission [42]. Copyright 2020, ACS

With the improvement of magnetic field generation equipment and the enrichment of magnetic signal types, more possibilities for magnetically responsive neuromodulation have been provided. For example, physical signal converted from magnetic field does not act directly on the ion channel, but reacts with the microenvironment of target cell, giving rise to a new subarea called magnetically induced microenvironmental-responsive neuromodulation. In fact, the influence of magnetic field and other signals transformed from it on nerve cell microenvironment have been explored recently independent from NNM. Jiang et al. [72] clarified that repeated magnetic field could improve the microenvironment of neural regeneration in spinal cord, such as reducing apoptotic cells, decreasing the expressing level of genes impeding spinal cord repair like matrix metalloproteinase 9/2, recovering body sensation and motor-induced currents, and eventually recovering motor function (Fig. 10a). Similar potential has also been discovered in physical signals converted from magnetic field, like the use of magneto-mechanical induced mechanochemical stimulation to modulate the folding and complex unbinding of proteins relevant to the treatment of SCI [73].

Typical magnetically induced microenvironmental-responsive neuromodulation was firstly achieved based on magnetothermal effect. The cooperation between AMF and MNPs not only results in local temperature improvement but also further affects thermal-sensitive biochemical reactions in certain microenvironments, providing additional application of this type of signal in neuromodulation. Park et al. [42] utilized such temperature rise to facilitate polyanhydrides and polyesters’ hydrolysis, where -COOH would be produced to less invasively lead to modulation of pH outside the cells and the activation of acid-sensing ion channel (ASIC) [74] (Fig. 10b). They designed MNP-PSA and MNP-PLGA nanosystems, covering Fe_3_O_4_ MNPs with poly(sebacic acid) (PSA) and poly(lactic-*co*-glycolic acid) (PLGA) scaffolds, respectively. In the Tyrode's solution irradiated with AMF containing MNP-PSA, a significant pH drop occurred especially in the first minute (Fig. 10c). In vitro experiment on ASIC1a^+^ nerve cells evidenced that MNP-PSA with AMF group resulted in obviously higher percentage of calcium influxes (expressed by the proportion of Ca^2+^-stimulated neurons), offering the potential to lead to neural excitation (Fig. 10d). In addition, they analyzed pH as well as temperature changes in the buffer system mimicking the MNP-PSA treatment of human cerebrospinal fluid. And it was observed that the pH value performed an obvious decrease followed by gradual recovery along with the on–off of AMFs only in the center of injection sites, while hardly any temperature rise occurred in not only the center but also the surroundings, suggesting the potential of this method to treat nerve cells rich in ASIC without causing nonspecific temperature changes (Fig. 10e). This magnetically responsive modulating technique not only facilitated the remote control of proton-regulated neural activities, but also provides a universal method for analyzing other proton-regulated bodily processes.

In a word, contributed by the intrinsic advantages of magnetic field such as high penetration depth and non-invasiveness, and the rapid development of magnetic-responsive functional nanoparticles, magnetic-responsive neuromodulation will play an increasingly important role in the regulation of neuronal behavior and the treatment of neurological diseases by converting magnetic fields into various external field signals or further interacting them with intracellular environments.

## Ultrasound-responsive neuromodulation

**Fig. 11:**
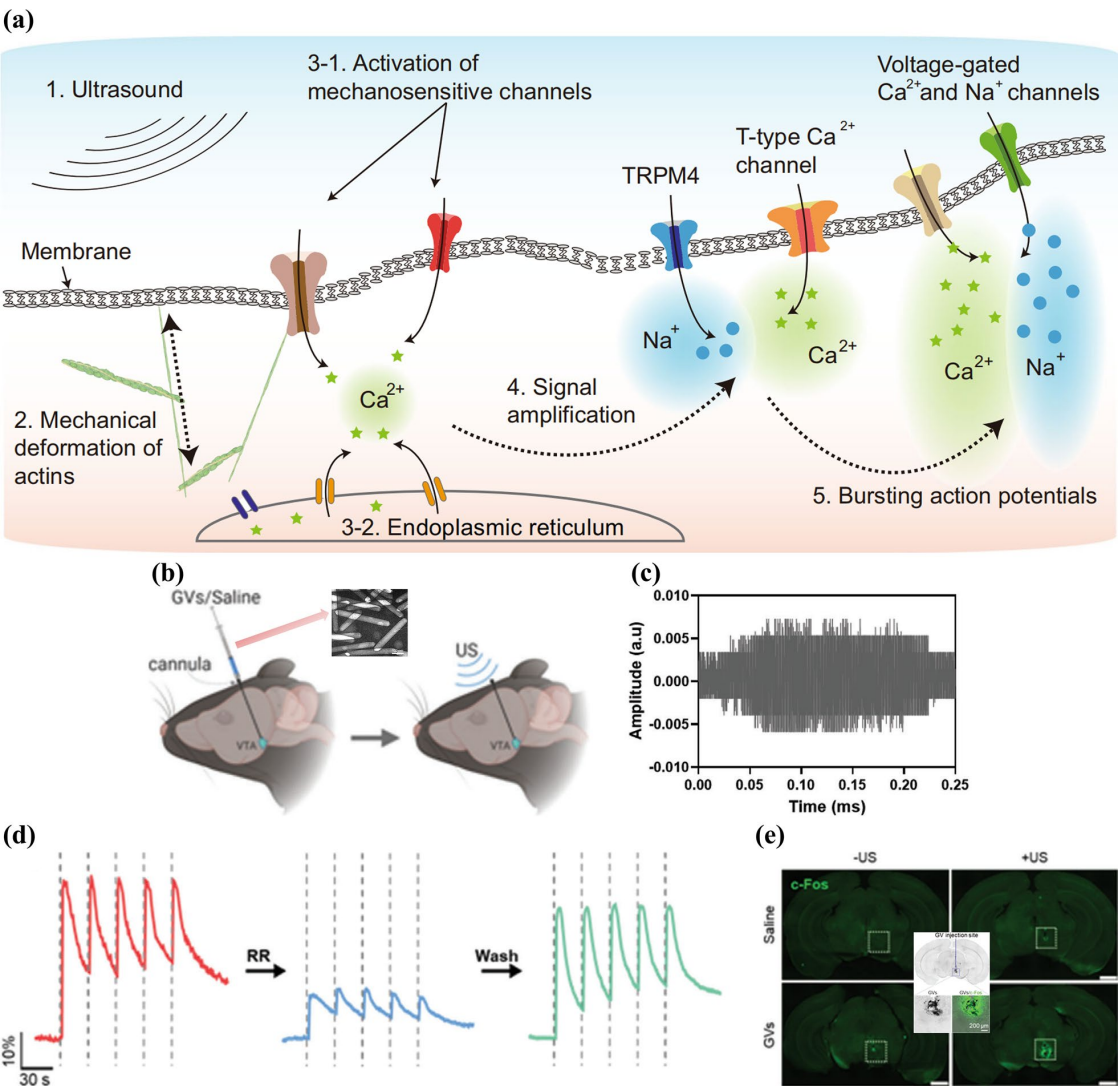
**a** Schematic diagram showing the ion channel processes triggered by ultrasound. Reprinted with permission [14]. Copyright 2022, Springer Nature. **b** The operation process of injecting GVs and applying ultrasound to mice brains. Inset is the TEM image of GVs. **c** Backscattered signals of GVs irradiated by US (1.0 MHz, 0.28 MPa). **d** Nerve Ca^2+^ influx degrees stimulated by US and GVs before, during and after the existence of ruthenium red. **e** Representative images of *c-Fos* expression level in differently treated mice brains, where GVs injection areas of VTA are inside the white dashed boxes. Inset is the comparation of GVs and *c-Fos* distribution. Reprinted with permission [44]. Copyright 2021, AAAS

Although ultrasonic signals have performed the potential to be non-invasively delivered into deep brain with well spatial accuracy, ultrasound-responsive approach used to be a less involved branch in NNM, partly because their modulatory mechanisms have not been clearly and entirely illustrated until recently [75, 76]. Previous researches only clarified that ultrasound could activate mechanosensitive ion channels like Piezo1 [77], TREK-1/2 [78], and TRPA1 [79] without elucidating the overall modulation pathway of ultrasound on neural activity until the study of Yoo *et al* [14] in 2022. According to them, the strategy of ultrasound action on neuromodulation consists of the following steps, (1) Ultrasound acts on the cell membrane will causes mechanical deformation of actin, which will trigger the release of Ca^2+^ from mechanosensitive ion channels and endoplasmic reticulum to the cytoplasm; (2) the increase of Ca^2+^ will further promote the influx of Na^+^ and Ca^2+^ from TRPM4 and T-type calcium channels, respectively, to amplify the original signal; and (3) The amplified signal will finally induce voltage-gated calcium and sodium channels, which ultimately produce action potential (Fig. 11a) [14]. Followed by this breakthrough, focused ultrasound signal has been applied to the modulation of some neurological diseases or processes, like diabetic autonomic pathways [80], long-lasting hippocampal synaptic plasticity [81], and torpor-like hypothermia [82]. However, these direct ultrasound modulation strategy are based on large-scale medical equipment rather than functional nanomaterials, and the required penetration and precision of the ultrasound signal are conflicting, with low-frequency ultrasound facilitating penetration of the skull while achieving nanoscale precision modulation requires higher frequencies, which is a major obstacle to direct neuromodulation with ultrasound [14, 44]. Therefore, in order to combine both high penetration and single-cell accuracy, indirect approaches relying on the transformation from ultrasound that has penetrated the barrier to other signals like the local focusing of ultrasound in the form of mechanical vibration [44], or ultrasound-electric transformation [83], have become the main current and both of which rely on functionalized nanoparticles to induce [84].

### Ultrasound-mechanical responsive neuromodulation

The propagation of sound in a medium is itself a kind of mechanical wave, so the conversion between ultrasonic and mechanical signals, also called the focus of ultrasound, was the first to be applied to neuromodulation and could be regarded as direct ultrasound-responsive approach to a considerable extent, by triggering the release of Ca^2+^ from mechanosensitive ion channels [85]. Hou et al. [44] achieved precise deep brain modulation by enhanced and targeted ultrasound signals in virtue of a nanoscale gas vesicle (GV) (Fig. 11b). GV refers to a cyanobacteria-produced hollow protein structure which could generate non-linear acoustic oscillations by the buckling effect of ultrasound. This secondary signal could transmit and enrich mechanical vibrations like microbubbles to nanoscale surroundings of GVs, thus lower the intensity requirement and increase the spatial resolution of ultrasound stimulation. They extracted GVs from *Anabaena flos-aquae* and exposed them to US of 0.28 MPa (much lower than the critical collapse pressure of GVs). And no broadband and only harmonic signals were detected (Fig. 11c), which suggested the cavitations produced by GVs refers to the stable type (only makes size transformation of bubbles) rather than inertial one (corresponds to stronger acoustic signals, damaging bubbles and harming cells), indicating the stability and safety of GVs. Then they applied US to nerve cells in GVs-enriched areas and detected immediate and reversible calcium influx, which could be distinctly but not completely weakened by ruthenium red (RR, the mechanosensitive ion channel blocker) and will recover by washing RR away (Fig. 11d). Therefore, a crucial stimulating mechanism of GVs + US should be the triggering of mechanosensitive ion channels, which acts as the crucial ion channel in the first step of the strategy of ultrasound action on neuromodulation. Furthermore, they evaluated the expressing level of *c-Fos*, a marker of calcium influx-caused nerve action, in GVs + US-treated VTA by implanting a cannula to administrate GVs. It was found that the distribution of activated neurons (bright green) was highly consistent with GVs (dark areas of brightfield images) (Fig. 11e). In a word, GVs enabled precise and non-invasive neuromodulation in mouse VTA by focusing on the triggering of mechanosensitive ion channels using low intensity US signals and performed the potential to achieve practical ultrasound responsive neuromodulation in human brains.

### Ultrasound-electric responsive neuromodulation

**Fig. 12:**
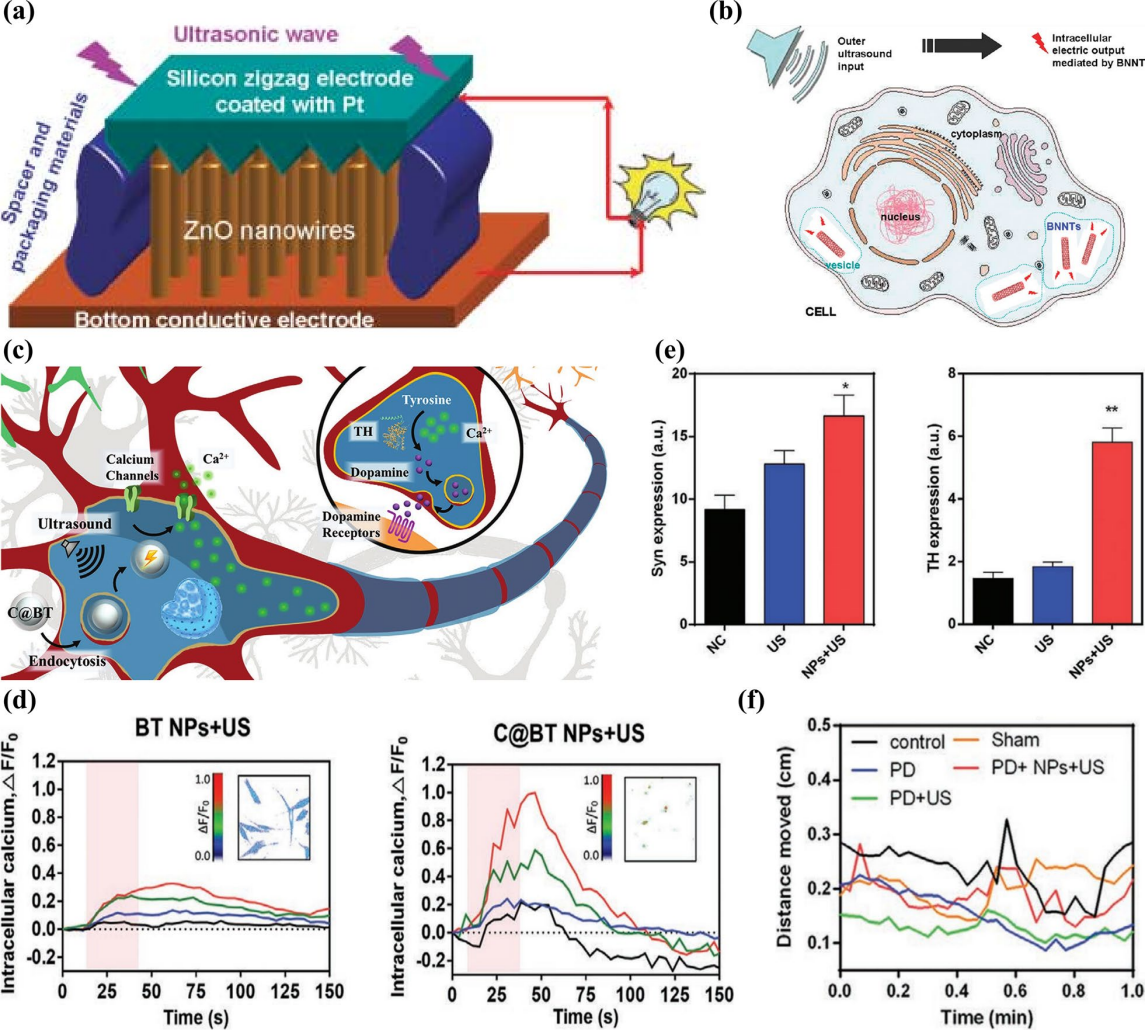
**a** Schematic illustration of a nanoscale ultrasound-triggered electricity generator. Reprinted with permission [86]. Copyright 2007, AAAS. **b** Exogenous ultrasound-triggered remote electrical cell control achieved by piezoelectric nanoparticles. Reprinted with permission [87]. Copyright 2010, ACS. **c** Schematic illustration of electromagnetized-nanoparticle-mediated neuron plasticity. **d** Comparison of fluorescence intensity variations of Ca^2+^ between two groups, where the ultrasound-stimulated period was in light red. Inset is a typical image showing maximal-changed intensity of fluorescence. **e** Comparison of the expression level of two genes (Syn and TH) among three groups. **f** The distance moved every 300 ms of PD zebrafishes in different groups. Reprinted with permission [83]. Copyright 2020, Wiley–VCH GmbH

The convenient transformation to electric signals is a remarkable advantage of ultrasound, and this conversion process has been realized in nanoparticle for a long time. For example, Wang et al. [86] designed a nanoscale ultrasound-triggered electricity generator device that consists of a vertical matrix of ZnO nanowires under a zigzag electrode. When irradiated with ultrasound, the electrode followed by the nanowires will mechanically vibrate and then transform mechanical signal to electricity through a piezoelectric effect. And its application in the field of neurology has also been attempted (Fig. 12a). Ciofani et al. [87] took advantage of piezoelectric boron nitride nanotubes (BNNTs) to convert exogenous ultrasound-induced mechanical vibration into electricity, which can promote neurite growth in neuron-like cells (Fig. 12b).

Nowadays, NNM based on ultrasound-electric conversion has emerged. A particular range of electromagnetic fields (EMF) can influence ion transport, intracellular communication and neuron activity [85]. The nanoparticles with ultrasound-electric effect could combine exogenous ultrasound with the EMF, which is a representative example of ultrasound-electric responsive neuromodulation. Zhao et al. [83] prepared a nanoparticle with core–shell structures (C@BT) by assembling piezoelectric BaTiO_3_ NPs and specially-made carbon shells, which can serve as a wireless approach to cure PD under the electromagnetization of ultrasound stimulation (Fig. 12c). They monitored the flow of Ca^2+^, a modulator of neuroplasticity, to the cells when exposed to the EMF generated by ultrasound. And according to Fig. 12d, the C@BT-treated group performed the highest Ca^2+^ influx intensity compared to the other groups. Then, they used immunofluorescence to analyze the effect of C@BT on the expression of synaptophysin (Syn) and tyrosine hydroxylase (TH), which serve as indicators of synaptic plasticity and dopamine (whose degradation is associated with PD), respectively, during the regeneration of dopamine neurons.

When exposed to ultrasound, the expressing level of Syn and TH clearly improved in C@BT group, which demonstrated in a molecular biological level that this approach combines the function of modulating neural plasticity with activating degenerative dopamine neurons (Fig. 12e). Furthermore, the effect of PD treatment in zebrafish was evaluated by C@BT with the help of an automated quantified behavior analysis system. It was found that after 5 days of treatment with C@BT NPs, the mobility of PD zebrafish was significantly recovered compared to the PD and PD + US groups, as shown by longer movement time and longer movement distance within the same time (Fig. 12f). Hence, the EMF-regulated nanoparticle system gives hope to achieving a minimally invasive and wireless method to cure neurodegenerative diseases by modulating synaptic plasticity and recovering degenerative dopamine neurons.

In summary, although the contradiction between penetration and accuracy of ultrasound signal makes it difficult to directly realize nanoscale neuromodulation, the rich transformation types between ultrasound and other signals make indirect ultrasound-responsive neuromodulation practical. In the future, in addition to as reported ultrasound-mechanical and ultrasound-electric responsive methods, ultrasound-thermal as well as ultrasonic catalysis, which have been applied in other fields, are also worth trying in NNM. Moreover, since the direct modulation pathway of ultrasound has been elucidated, another category of indirect neuromodulation approaches is also worth exploring, i.e., the conversion of other highly penetrating signals into ultrasound in target cells by nanoparticles, such as photoacoustic neuromodulation [84, 88].

## Self-powered mechanical-responsive neuromodulation

As mentioned above, traditional direct electrical neuromodulation methods lack spatial accuracy and are prone to induce infection due to their invasiveness. Although NNM methods such as photoelectric, magnetoelectric, and ultrasound-electric responsive neuromodulation have achieved non-invasive and precise nerve regulation, corresponding external field signal generation devices as new medical equipment have to be introduced. On the other hand, mechanical stretching generated by the movement of the human body itself can be self-provided to generate electrical signals with the help of nano-piezoelectric devices, so as to realize spatially accurate indirect electrical-responsive neuromodulation without applying external field signals [89]. Such self-powered mechanical-responsive neuromodulation may represent another strategy of NNM.

**Fig. 13:**
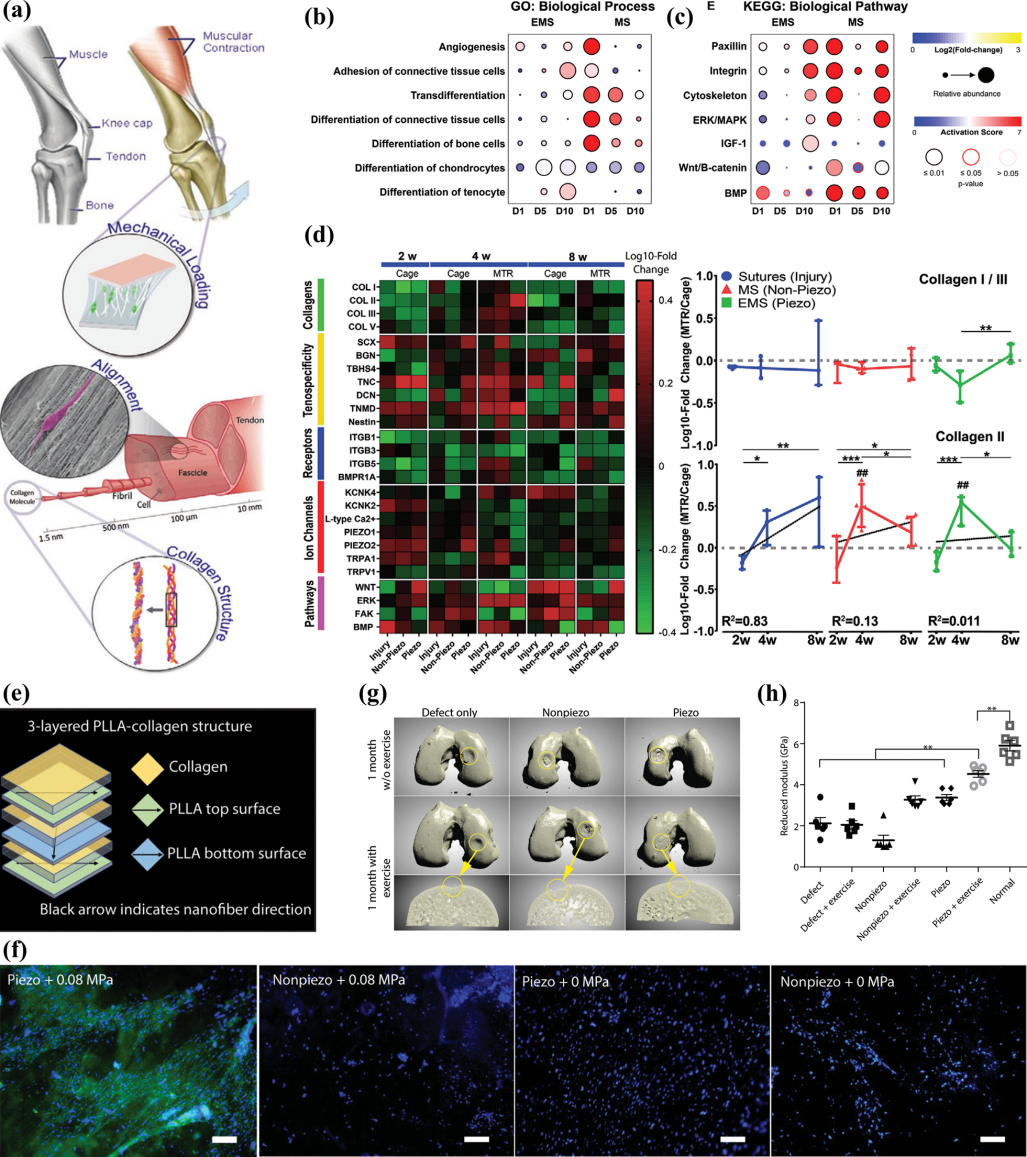
**a** Schematic illustration showing the electromechanical constructions of tendons. Obvious differences between tendon cells stimulated by MS and EMS signals in expressing gene operating **b** certain bioprocesses and **c** signal channels. **d** The microarray heatmap indicating the variation of expressing specific protein in different groups. Reprinted with permission [24]. Copyright 2021, Wiley–VCH GmbH. **e** Schematic diagram showing the structure of the 3-layered PLLA nanofiber scaffold with piezoelectricity and biodegradability. **f** Immunofluorescent images of type II collagen in green as well as cell nucleus in blue of ADSCs with different treatments. **g** Micro-CT reconstructed images showing differently-treated subchondral bone regeneration in rabbits. **h** Reduced modulus differences in the interfaces of regenerated rabbit cartilages with different treatments. Reprinted with permission from [45]. Copyright 2022, AAAS

Based on the mechanically sensitive ion channels in tendon cells and the distinct possibility of electrical fields to heal wound, self-powered mechanical-responsive neuromodulation, with its aim at activating in vivo signal channels for repairing tissue, represents the change of research directions in areas of biomedical equipment as well as regenerative medicine (RM). Fernandez-Yague et al. [24] applied piezoelectric collagen-like PVDF-TrFE, a scaffold composed of directional nanofibers made from ferroelectric material poly(vinylidene fluoride-*co*-trifluoroethylene), to non-tendinous tissue recovery assisted by electromechanical (EMS) signals (Fig. 13a). The influence of EMS + PVDF-TrFE on expressing human TDCs (hTDCs) gene and generating protein were observed at several time nodes on human genome analysis, which were statically and dynamically loaded in physiological-related conditions. The result observed at the level of gene is closely related to that of protein, suggesting that EMS can regulate hTDCs trans-differentiation into osteoblast or chondrocyte lines and has the ability of sustainedly promoting the tendon cell differentiation compared with mechanical stimulation (MS) (Fig. 13b, c). Furthermore, the effect of EMS + PVDF-TrFE scaffolds in vivo was evaluated using a custom protein array, which was applied to estimate collagen generation, tendon specificity, expression of cellular mechanoreceptors, ion channels and signal pathway activation. It was proved that EMS regulates the expression of mechanically sensitive ion channel bone morphogenetic protein receptor (BMP) and modulates its activating time and level, thus affecting the tendon specific tissue recovery (Fig. 13d). This research showed that in the process of self-powered mechanical-responsive neuromodulation, electromechanical signal is able to modulate the sensitivity of mechanically sensitive ion channel and improve tendon specificity during non-tendinous tissue recovery.

Self-powered mechanical-responsive neuromodulation also has the potential to achieve synergistic effect with healthy exercise, not just simple body movement. Liu et al. [45, 90] combined piezoelectric poly(l-lactic acid) (PLLA) nanofibers with physical training to produce beneficial surface nanoampere current, which will contribute to the recovery of hyaline cartilage, to achieve improvement in the therapy of osteoarthritis (OA). They packaged 3 films of PLLA, where the nanofibers were lined up in uniform directions respectively, with collagen layers, which acts as binders and space for the growth of cells, to establish a three-dimensional 3-layered biodegradable piezoelectric PLLA nanofiber scaffold (Fig. 13e). Immunofluorescent images of type II collagen proved that the application of mechanical signals to scaffold-cultured rabbit adipose-derived stem cells (ADSCs) promoted the growth of type II collagen (Fig. 13f). Further research on the knee joints of rabbits conformed that scaffold-treated and exercise-induced samples regenerated subchondral bone the most effectively and achieved recovery in reduced modulus (Fig. 13g, h). In summary, by cooperating with rehealthy training, this piezoelectricity responsive and biodegradable neuromodulation method will enhance differentiation into cartilage cells and help regenerating cartilage, highlighting its application value for OA treatment.

To sum up, self-powered mechanical-responsive neuromodulation has developed a unique path different from the aforementioned light, magnetic and ultrasound responsive methods, by replacing additional applied external physical fields with mechanical signals generated by the human's own motion. In the future, with the gradual miniaturization of nanodevices, the spatial resolution of self-powered mechanical-responsive neuromodulation will be further improved. Furthermore, by introducing nanomaterials that can convert mechanical force into more signal types besides electricity, self-powered mechanical-responsive neuromodulation will play roles in the regulation of more types of neural activities and the treatment of related diseases.

## Conclusions

Thanks to the rapid progress of nanomaterials and neurobiology, various types of stimuli-responsive nanoparticles targeting ion channels in nerve regulation have been developed, which greatly promote the advances of non-genetic NNM, a newly emerging multi-disciplinary field. This state-of-the-art research hotspot, concentrating on the recent advances in neuro and material science, has been offering more and more possibilities to the effective therapeutics of neurological disorders as well as the understanding of the deep running mechanism of human nervous system. As have been reviewed above, non-genetic NNM has now grown into a multi-disciplinary field with various branches, based on the light, ultrasound, electric current, magnetic field and mechanical stimulations, etc., by modulating relevant ion channels and employing specific nanoparticles, which provides novel technologies for the treatment of the nervous system diseases (Table 1). However, such a functional inorganic nanoparticle enabled non-genetic neuromodulation is still in its infancy of development, and there are still a number of challenges to be solved for future development, including the rational designs of bio-nanomaterials with diversified external field-responsive functions, the mechanism probing of neuro-stimulations and nanoparticles’ targeting effect toward signal pathways, and the long-term biological effects and safety evaluations for clinical applications.

### Overcoming technological barriers of exogenous physical signals

The exploration of the regulatory mechanisms over the actions of unknown pathways triggered by exogenous physical signals, along with relevant comprehensive knowledge of multiple disciplinary fields and experimental techniques from material preparation to behavioural analysis, are the apparent challenges in the non-genetic NNM research, which necessitates the joint efforts from diverse researches to overcome the barriers. And due to the different degree of the above barriers for signal types should overcome, they show disparate development status in NNM, where ultrasound, light irradiation and magnetic field are widely applied while applications of electricity, mechanical force and multili-signals are still in the initial stage. Furthermore, the simple, less invasive and targeting effects are of importance in designing and preparing non-genetic NNM in most biological applications of nanoparticles. In order to achieve these goals, factors such as the properties of exogenous signals, the performances of nanoparticles and signal conduction and transformation pathways, should be carefully considered, selected and integrated. These requirements bring both opportunities and challenges. For example, ultrasonic signals perform excellent spatial targeting effects and penetration depth, and its in-depth mechanism of neuromodulation is nowadays under vigorous investigations, which makes this field highly promising and worth exploration [14].

**Table 1:** Summary of the signals, transformation paths, ion channels, nanomaterials and diseases or processes involved in non-genetic nano-neuromodulation

Signal types	Transform paths	Ion channels involved	Typical materials	Disease/process examples
Light irradiation	Photothermal	TRP and TREK	Photothermal nanoparticles	Atherosclerosis
	Photoconversion	ChR2	Upconversion nanoparticles	Sensing activities
	Photochemical-catalytic	CRAC	Upconversion nanoparticles	Traumatic spinal cord injury, stormorken syndrome
	Photoelectric	voltage-gated calcium channels	Photoelectric nanocomposites	Epilepsy
	Photomechanical	Piezo1, Piezo2	Optomechanical nanoparticles	Molecule movement, cell displacement and activation
Magnetic field	Magnetothermal	TRPV1	Superparamagnetic nanoparticles Magnetothermal nanoparticles	Wireless deep brain stimulation
	Magneto-mechanical	Piezo1	Magneto-mechanical nanoparticles	Motor function
	Magnetoelectrical	voltage-gated calcium channels	Core–shell magnetostrictive piezoelectric nanoparticles	wireless deep brain stimulation
	Magnetically induced microenvironmental	ASICs	Magnetothermal nanoparticles	Spinal cord injury, proton regulated neural activities
Ultrasound	Ultrasound-mechanical	Mechanosensitive ion channels	Nanoscale gas vesicles	Precise deep brain modulation
	Ultrasound-electric	voltage-gated calcium channels	Piezoelectric nanoparticles	Parkinson’s disease
Mechanical force	Self-powered mechanical	Mechanically sensitive ion channels	Piezoelectric nanofibers	Wound heal, osteoarthritis

### Design, preparation and expansion of functionalized nanomaterials

A number of nanoparticles have been found to perform desired signal adsorption and/or conversion capability like gold nanorods in photothermal modulation, while others like core-shell structured magnetostrictive-piezoelectric nanoparticles in ultrasound-electricity transformation are the combination of two or more types of nanoparticles with different signal transforming abilities [37, 43]. These thoughts of nanoparticle designs are not versatile and feasible enough in developing ever-specialized nanoparticles for diversified applications. However, due to the great physical variations among different external signal types, more attentions should be paid to the exploration of multi-functional nanoparticles specifically applicable in certain sub-area of non-genetic NNM rather than the whole field. Certain nanoparticles have already performed the potentials to serve as general platforms in the relevant NNM categories, such as UCNPs in light-responsive neuromodulation. Through modifying strategies like the dope of rare-earth elements, the cooperation with other functional components and the modifications using antibodies or other identification proteins, UCNP can not only achieve various types of upconversions, but also play roles in photothermal processes and photo-induced neurotransmitter (like NO) release, suggesting that this nanomaterial is promising to serve as a general platform. Such a methodology of multi-component integration among nanoparticles and modulative biological proteins is of significance and values, and new opportunities are still available in these field for the development of advanced material preparation processes, nevertheless, innovative nanotechnology and nanomaterials, and the discovery of energy conversion mechanisms, are more desirable, which necessitates more extensive and interdisciplinary efforts.

Another developing trend of materials applied in neuromodulation is the extension of material types from inorganic nanoparticles to hybrid organic–inorganic materials, and even purely organic systems, such as microbiological products and macrodevices [91], which are even out of the nanomaterial category. Due to their excellent function of transforming and transmitting physical signals, inorganic nanomaterials have been the pioneer nanosystems applied to NNM. However, the poor biocompatibility and targeting of inorganic nanomaterials also limited the in-depth research and clinical translation of NNM, thus it is necessary to combine them with organic nanostructures like biocompatible organic groups or antibodies to form organic–inorganic hybrid nanoparticles [92]. Moreover, pure organic nanoparticles have also managed to secure a place especially in physical field-chemical responsive strategies of NNM, such as photochemical-catalytic methods, where specific biochemical reactions instead of physical signal conversion occurs in materials. Another advantage of pure organic nanoparticles should be the convenience of forming macrodevices, like PVDF-TrFE [24] and PLLA [45] structures in self-powered mechanical-responsive neuromodulation. And the shortcoming of organic nanoparticles in the poor functionality of transforming physical signals could be overcome by some microbiological products (e.g., GVs [44]) in ultrasound-mechanical strategy. In summary, organic–inorganic hybrid nanoparticles, which combines the functionality of inorganic nanoparticles with the biocompatibility and targeting effect of organic nanoparticles, will be the mainstream of NNM, and pure organic systems like microbiological product and macrodevices, will also become a new entry point for research.

### Clinical translation orientation

Strictly speaking, the existing long distance of the non-genetic NNM research to clinical test is not surprising for a novel area. However, the underlying causes of this situation should be clarified to accelerate the clinical translations. As a matter of fact, such a long distance is the result of mutual interactions among multiple factors including the technique barriers discussed above, as well as the difficulty of the transition from animal models to human experiments. Therefore, even more strict requirements have been put forward based on the factors just mentioned above. On the one hand, human trials should be actively conducted under the strict monitoring of safety and compliance. On the other hand, the repeatable and large-scale preparation of modulative nanoparticles could be paid more attentions based on the available and novel modulation strategies. Encouragingly, some developing trends gives us hope to overcome these technological barriers, such as the technical exchange and boundary fading between genetic and non-genetic NNM, which combines the research progress of the former for safety and the latter for functionality and promote the development and application of both branches.

**Fig. 14:**
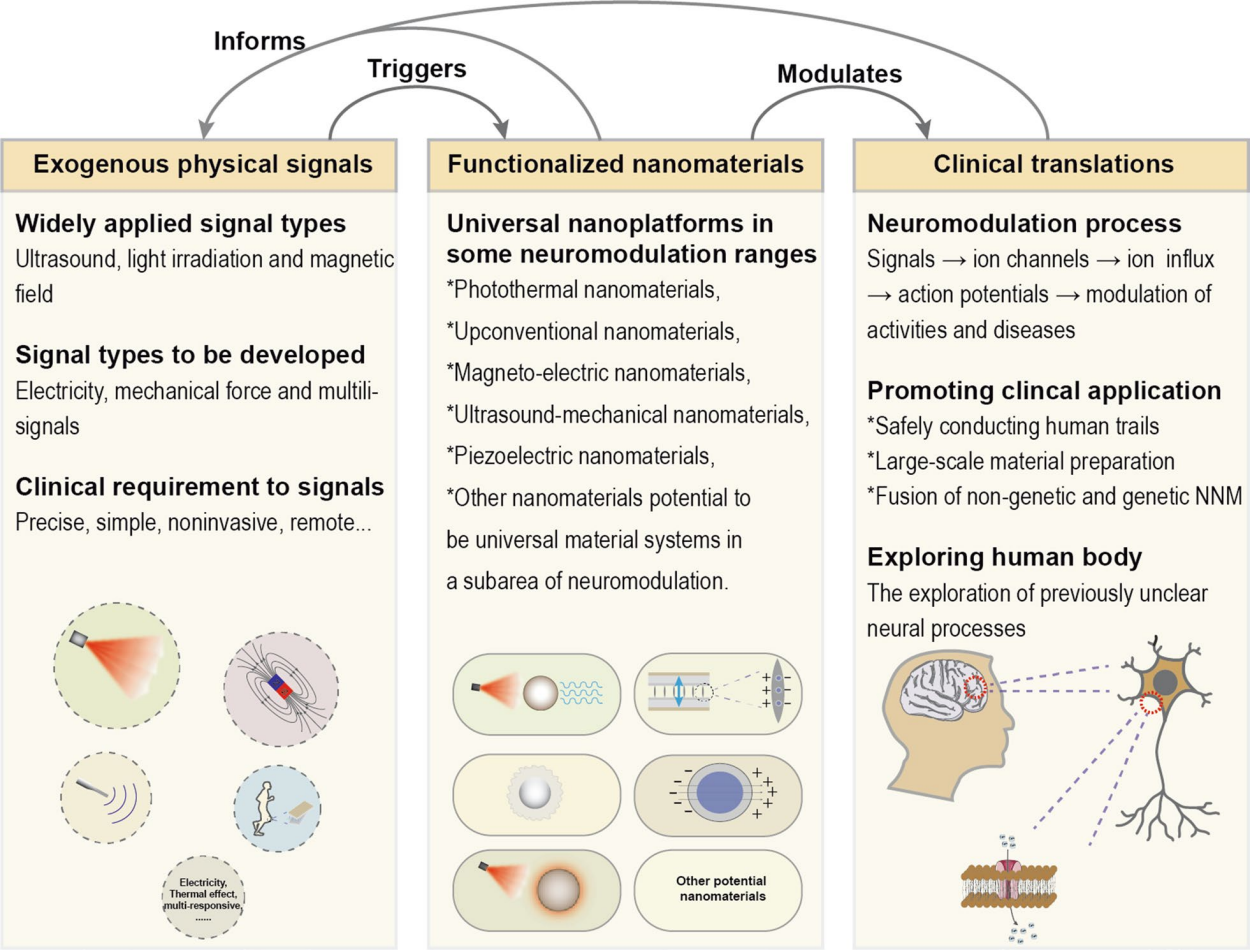
Conclusions and outlooks of non-genetic nano-neuromodulation development: The application status of different signal types and the clinical requirement to them; design and preparation of functionalized nanomaterials as universal nanoplatforms in some neuromodulation ranges; the clinical translation orientation of non-genetic NNM; as well as the connection route between signals, materials and nervous activities

Nowadays, the major attentions of non-genetic NNM are to be concentrated on the therapy of nerve diseases, where new findings of neurosciences and biology are the basis of novel neuromodulation methods and therapeutic treatments. However, clear understanding of previously unclear neural processes should be completed in clarifying the therapeutic mechanism of a neuromodulation method. For example, as described above, with the original aim of regulating the optical reactivity of polyps, Tortiglione et al. [23] have clarified pathways triggered by P3HT-NPs in optical spectra, and figured out the underlying mechanism of animal photosensitivity regulation. These findings, which might lead to breakthroughs in neuroscience, should not be viewed in an isolated manner or even ignored. Furthermore, because the nerve modulating approaches, such as the stimulation of ion channels and the release of neurotransmitters, are always acting on the deep-running process of nervous system, it is highly expected to explore the human neuron operating mechanisms in a more extensive and detailed manner using non-genetic NNM  techniques, in addition to the studies aiming at curing neurological deseases. Novel concepts in understanding the interactions among the nanoparticles, neuromodulations, external stimuli, etc., will be highly conducive to promote the development of both non-genetic NNM and neurobiology (Fig. 14).

To sum up, as an emerging and promising interdisciplinary fields, non-genetic NNM has been revealing great prospects in the neuromodulations and the therapeutics of various nerve diseases, some of which may be hardly treatable by using traditional methods. Based on the regulation of specific ion channels by exogenous stimuli through functionalized nanoparticles, non-genetic NNM performed great potentials to achieve simple, remote, non-invasive, and spatiotemporally precise treatments of neurological disorders. Although there are still numbers of challenges to be solved as a newly emerging field, non-genetic NNM is expected to develop more quickly and comprehensively in the near future as one of the major solutions in treating neurological diseases and understanding the operation mechanisms of the human nervous system.

## Data Availability

Not applicable.

## Abbreviations

NNM: Nano-neuromodulation
PD: Parkinson’s disease
AD: Alzheimer’s disease
HD: Huntington disease
ALS: Amyotrophic lateral sclerosis
SMA: Spinal muscular atrophy
TRP: Thermally sensitive transient receptor potential
ROS: Reactive oxygen species
GNRs: Gold nanorods
VSMCs: Vascular smooth muscle cells
oxLDL: Oxidized low-density lipoprotein
Cap: Capsaicin
UCNPs: Upconversion nanoparticles
UCL: Upconversion luminescence
UV: Ultraviolet
pbUCNPs: Photoreceptor-binding UCNPs
ERG: Electroretinogram
VEPs: Visual evoked potentials
VTA: Ventral tegmental area
ChR2: Channelrhodopsin-2
DA: Dopamine
SCI: Spinal cord injury
DRG: Dorsal root ganglion
ZIF-8: Zeolitic imidazolate framework-8
CysNO: Nitrosothiol
SOCE: Store-operated calcium entry
CRAC: Calcium release-activated calcium
PENCs: Photoelectric nanocomposites
P3HT-NP: Poly(3-hexylthiophene) nanoparticle
LED: Light-emitting diode
qRT-PCR: Quantitative reverse transcription polymerase chain reaction
PFC: Polymer force clamp
OMA: Optomechanical actuator
TIRF: Time-lapse total internal reflection fluorescence
TCRs: Receptors of T cells
RF: Radio-frequency
MNPs: Magnetic nanoparticles
AMF: Alternating magnetic field
m-Torquer: Magnetic torquer
CMA: Circular magnet array
*F*_*τ*_: Torque force
RT-PCR: PCR with reverse transcription
ACTB: β-Actin
MSNP: Magnetostrictive cobalt ferrite nanoparticle
MENP: Magnetoelectric nanoparticle
AC: Alternating current
DC: Direct current
DBS: Deep brain stimulation
ASIC: Acid-sensing ion channel
PSA: Poly(sebacic acid)
PLGA: Poly(lactic-*co*-glycolic acid)
GV: Gas vesicle
RR: Ruthenium red
BNNTs: Boron nitride nanotubes
EMF: Electromagnetic fields
Syn: Synaptophysin
TH: Tyrosine hydroxylase
RM: Regenerative medicine
PVDF-TrFE: Poly(vinylidene fluoride-*co*-trifluoroethylene)
EMS: Electromechanical stimulation
hTDCs: Human TDCs
MS: Mechanical stimulation
BMP: Bone morphogenetic protein receptor
PLLA: Piezoelectric poly(l-lactic acid)
OA: Osteoarthritis
ADSCs: Adipose-derived stem cells
